# Synthesis, biological evaluation, molecular docking, and MD simulation of novel 2,4-disubstituted quinazoline derivatives as selective butyrylcholinesterase inhibitors and antioxidant agents

**DOI:** 10.1038/s41598-024-66424-z

**Published:** 2024-07-06

**Authors:** Sara Sadeghian, Raziyeh Razmi, Soghra Khabnadideh, Mehdi Khoshneviszadeh, Pegah Mardaneh, Arman Talashan, Arman Pirouti, Fatemeh Khebre, Zahra Zahmatkesh, Zahra Rezaei

**Affiliations:** 1 Department of Medicinal Chemistry, School of Pharmacy, Shiraz University of Medical Sciences, Shiraz, Iran; 2 Pharmaceutical Sciences Research Center, Shiraz University of Medical Sciences, Shiraz, Iran; 3 Medicinal and Natural Products Chemistry Research Center, Shiraz University of Medical Sciences, Shiraz, Iran

**Keywords:** Quinazoline, Cholinesterase inhibitors, Antioxidant, Molecular docking, MD simulation, Chemical biology, Drug discovery, Diseases

## Abstract

Alzheimer’s disease is the most prevalent neurodegenerative disorder characterized by significant memory loss and cognitive impairments. Studies have shown that the expression level and activity of the butyrylcholinesterase enzyme increases significantly in the late stages of Alzheimer’s disease, so butyrylcholinesterase can be considered as a promising therapeutic target for potential Alzheimer’s treatments. In the present study, a novel series of 2,4-disubstituted quinazoline derivatives (**6a**–**j**) were synthesized and evaluated for their inhibitory activities against acetylcholinesterase (AChE) and butyrylcholinestrase (BuChE) enzymes, as well as for their antioxidant activities. The biological evaluation revealed that compounds **6f**, **6h**, and **6j** showed potent inhibitory activities against eqBuChE, with IC_50_ values of 0.52, 6.74, and 3.65 µM, respectively. These potent compounds showed high selectivity for eqBuChE over eelAChE. The kinetic study demonstrated a mixed-type inhibition pattern for both enzymes, which revealed that the potent compounds might be able to bind to both the catalytic active site and peripheral anionic site of eelAChE and eqBuChE. In addition, molecular docking studies and molecular dynamic simulations indicated that potent compounds have favorable interactions with the active sites of BuChE. The antioxidant screening showed that compounds **6b**, **6c**, and **6j** displayed superior scavenging capabilities compared to the other compounds. The obtained results suggest that compounds **6f, 6h,** and **6j** are promising lead compounds for the further development of new potent and selective BuChE inhibitors.

## Introduction

Alzheimer’s disease (AD) is a progressive neurodegenerative disorder that gradually leads to memory loss, severe cognitive impairment, and behavioral and psychological symptoms^1,2^. Approximately 50–60% of dementia cases are caused by Alzheimer’s disease, and the number of these patients is expected to increase from 55 to 151 million by 2050^3^. Therefore, the development of new and improved therapeutic agents for the treatment of this disorder is crucial. The AD pathophysiology is complex and multifactorial, and various mechanisms have been suggested for its occurrence^1,4–6^. These mechanisms include cholinergic dysfunction, accumulation of amyloid-beta (Aβ) plaques, aggregation of tau proteins, reactive oxygen species (ROS) production, and inflammatory response^5–7^. Due to the complex nature of AD, studies have been directed towards multi-target-directed ligands (MTDLs) that act simultaneously on several targets involved in the disease, as a more efficient approach^3,8^.

**Figure 1:**
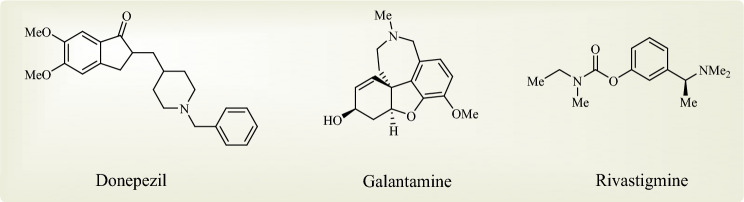
The chemical structures of the AChE inhibitor drugs currently used in Alzheimer's treatment.

According to the “cholinergic hypothesis” as the oldest hypothesis, Alzheimer’s disease is caused by the loss of cholinergic neurons and the subsequent gradual decrease in acetylcholine (ACh) levels in the forebrain, and one of the most effective strategies to improve the cognitive and behavioral symptoms of the AD is to prevent the decrease in acetylcholine levels^1,4,8,9^. Overall, the hydrolysis of ACh in the CNS is mainly carried out by acetylcholinesterase (AChE) and butyrylcholinesterase (BuChE) enzymes. Under normal conditions, AChE is about 10,000 times more involved in ACh hydrolysis than BuChE^10^. Hence, cholinesterase inhibitors such as donepezil, galanthamine, and rivastigmine (Fig. 1) by inhibiting the hydrolysis of acetylcholine and subsequently increasing the levels of ACh in neurons are effective in the treatment of Alzheimer’s disease^11,12^.

However, recent studies have shown that AChE levels decrease during the progression of AD, which leads to increased hydrolysis of ACh by BuChE. Under these conditions, BuChE may increase up to 165% of its normal physiological levels. Therefore, the inhibition of BuChE can be considered as a therapeutic target for the treatment of advanced-stage AD^1,13–16^.

AChE and BuChE are structurally similar enzymes, each containing a catalytic active site (CAS) and a peripheral anionic site (PAS) linked by a mid-gorge recognition site. The broader acyl-binding site of BuChE leads to recognition and hydrolysis of larger substrates. The structural features of BuChE facilitate the rational design of selective BuChE inhibitors with new scaffolds^17,18^. In recent years, several selective BuChE inhibitors have been developed, such as thiazole, tetrahydroacridine, 4-dimethylamine flavonoid, diarylinidazole, cymserine, benzofuran, and isosorbide-based derivatives. Despite the considerable efforts in this field, none of these compounds have progressed beyond in vitro studies, which emphasizes the need to discover and develop new selective BuChE inhibitors that can reach the drug candidate stage^1,19,20^.

It has been established that oxidative stress is also an important factor in AD pathogenesis. High levels of oxidative markers and antioxidant enzymes have been observed in patients with AD. Furthermore, it has been found that increased ROS secretion and impaired antioxidant defense may directly impair synaptic function and neurotransmission, leading to cognitive decline. Therefore, the development of cholinesterase inhibitors with antioxidant properties is an emerging trend in the design of new therapeutic agents for AD^21–24^.

The quinazoline scaffold, as a privileged structure, has been used for the design and development of various therapeutic agents, including anticancer, antimicrobial, and anti-neurodegenerative agents^25–27^. Recently, several quinazoline-based compounds have been reported as effective agents for the treatment of Alzheimer’s disease^28–30^. Among these, a number of 2,4-disubstituted quinazoline derivatives have shown effects such as cholinesterase and Aβ aggregation inhibition^31^. Mohamed et al. developed a library of 2,4-disubstituted quinazoline derivatives that exhibited significant cholinesterase inhibitory and antioxidant activity^25,31^. Considering the potential and effectiveness of 2,4-disubstituted quinazoline derivatives in Alzheimer’s disease, further studies on these compounds are necessary.

Here, we report the synthesis and biological evaluation of a novel series of 2,4-disubstituted quinazoline derivatives (**6a**–**j**) as selective BuChE inhibitors and antioxidant agents. These derivatives were obtained in high yields through the cyclization of anthranilic acid with urea, followed by the condensation reaction of the resulting 2,4-dichloroquinazoline intermediate with different anilines. The AChE/BuChE inhibition potencies of the synthesized derivatives **6a**–**j** were evaluated using the Ellman’s method, and their antioxidant activities were investigated using DPPH assay. Additionally, kinetic study, molecular docking, and MD simulation of these compounds were carried out to obtain information about the interaction type of these compounds in the active site of target receptor.

## Results and discussion

### Chemistry

**Figure 2:**
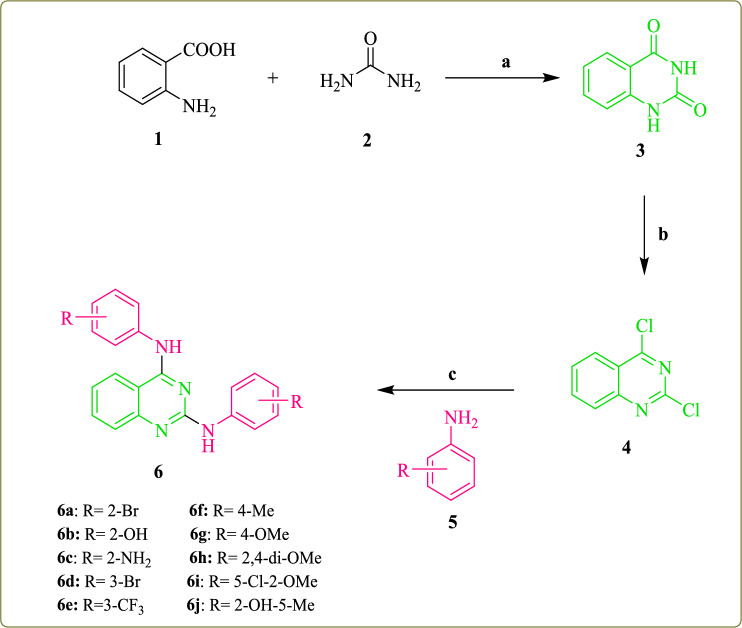
Synthesis of 2,4-disubstituted quinazoline derivatives **6a**–**j**. Reagents and conditions: (**a**) 190 °C, 24 h; (**b**) POCl_3_, *N*,*N*-diethylaniline, 115 °C, 16 h; (**c**) EtOH, reflux, 24 h.

The synthetic route of the quinazoline derivatives **6a**–**j** is represented in Fig. 2. In the first step, quinazoline-2,4-dione (**3**) was synthesized by cyclization of anthranilic acid with urea at 190 °C. In the second step, a mixture of quinazoline-2,4-dione (**3**)**,** phosphoryl chloride (POCl_3_), and N, N-diethylaniline, was refluxed at 115 °C for 16 h to give 2,4-dichloroquinazoline (**4**). In the final step, quinazoline derivatives **6a-j** with various substituted phenyl at the C-2 and C-4 positions were prepared through the condensation reaction of 2,4-dichloroquinazoline (**4**) with different anilines (**5**) in ethanol under reflux conditions, with 68–85% yields. The structures of the novel synthesized compounds have been confirmed by means of FT-IR, ^1^H-NMR, ^13^C-NMR, and Mass spectroscopy techniques (see supplementary files).

In the FT-IR spectra of the synthesized compounds, a stretching band at 3188–3449 cm^−1^ confirmed the presence of NH groups, the stretching bands at 1474–1568 cm^−1^ indicated the presence of aromatic rings, and the stretching band at 1584–1639 cm^−1^ showed the presence of C=N groups. In the ^1^H-NMR spectra of the synthesized compounds, two singlet signals at 10.78–11.64 ppm and 9.38–10.86 ppm confirmed the presence of two NH groups, which are characteristic signals and confirmed the structure of synthesized compounds. The OH groups in the structures of compounds **6b** and **6j** appeared at 8.20–9.33 ppm as a singlet signal. The two CH_3_ groups in the structures of compounds **6f** and **6j** appeared at 1.95–2.36 ppm as a singlet signal, while the OCH_3_ groups in the structures of compounds **6g**, **6h**, and **6i** appeared at 3.76–3.85 ppm as a singlet signal. The other hydrogens associated with the phenyl rings and quinazoline ring were observed at the expected chemical shifts. In the ^13^C-NMR spectra, two peaks at 150–160 ppm confirmed the presence of C-2 and C-4 of the quinazoline ring. The signal of methyl group in compounds **6f** and **6j** appeared in the region around 20 ppm, while the signal of methoxy groups in compounds **6g**, **6h**, and **6i** appeared at 50–60 ppm. Signals corresponding to other carbons in the structures appeared at the expected chemical shifts.

### Cholinesterase inhibitory

The inhibitory potencies of the 2,4-disubstituted quinazoline derivatives **6a**–**j**, along with donepezil as the standard drug, against electric eel acetylcholinesterase (eelAChE) and equine serum butyrylcholinesterase (eqBuChE) were evaluated according to the Ellman’s method, and the obtained results are summarized in Table 1.

**Table 1:** The inhibitory activities of the synthesized compounds (**6a**–**j**) towards eelAChE and eqBuChE.


Compounds	R	eelAChE: IC_50_ (µM)^a^	eelAChE: % inhibition^b^	eqBuChE: IC_50_ (µM)^a^	eqBuChE: % inhibition^b^	Selectivity index to BuChE^c^ (SI)
6a	2-Br	> 50	12.45 ± 2.74	> 50	30.64 ± 2.15	–
6b	2-OH	> 50	8.7 ± 1.15	37.23	52.3 ± 2.17	–
6c	2-NH_2_	> 50	6.15 ± 3.15	26.85	64.08 ± 2.32	–
6d	3-Br	> 50	23.49 ± 1.03	41.75	56.14 ± 4.17	–
6e	3-CF_3_	> 50	16.50 ± 3.05	43.54	58.75 ± 1.37	–
6f	4-Me	> 50	40.34 ± 3.55	0.52	96.74 ± 2.57	> 96
6g	4-OMe	> 50	11.04 ± 4.64	48.34	51.48 ± 3.25	–
6h	2,4-di-OMe	47.65	52.43 ± 2.33	6.74	87.47 ± 3.08	7.1
6i	5-Cl-2-OMe	> 50	13.67 ± 1.94	> 50	26.64 ± 3.19	–
6j	2-OH-5-Me	> 50	45.25 ± 2.91	3.65	91.63 ± 3.26	> 13.6
Donepezil	–	0.027 ± 0.01	80.97 ± 1.90	10.6 ± 2.1	86.53 ± 2.07	–

^a^IC_50_: 50% inhibitory concentration (mean ± SD of three independent experiments).

^b^%inhibition at 50 µM concentration for eelAChE and eqBuChE, respectively.

^c^Selectivity index to BuChE (SI): IC_50_ for AChE/ IC_50_ for BuChE.

From the obtained results it has been cleared that the synthesized compounds show higher potency towards eqBuChE rather than eelAChE. Among all the synthesized compounds, compounds **6f**, **6h**, and **6j** showed the highest inhibition percentage of eelAChE with 40.34%, 52.43%, and 45.25%, respectively. Compound **6h** with two methoxy substitutions at the *ortho* and *para* positions of the phenyl rings (2,4-di-OMe), displayed the most activity against eelAChE with an IC_50_ value of 47.65 µM. These results show that the introduction of one methyl group (**6f** and **6j**) or two methoxy groups (**6h**) on the phenyl ring improves the inhibitory activity against eelAChE.

Compounds **6f**, **6h**, and **6j** also indicated the highest activities towards eqBuChE, with IC_50_ values of 0.52, 6.74, and 3.65 µM, respectively. As observed, compounds **6f**, **6h**, and **6j** exhibited high selectivity for eqBuChE over eelAChE. Compound **6f** with a 4-methyl (4-Me) substitution at the phenyl rings, exhibited the highest inhibitory activity against eqBuChE with an IC_50_ value of 0.52 μM and a selectivity index (SI) of > 96 for eqBuChE over eelAChE. Interestingly, eqBuChE inhibitory activity displayed by compound **6f** was 20 times more active than donepezil with IC_50_ value of 10.6 μM.

In recent years, a number of quinazoline derivatives have been designed and synthesized as cholinesterase inhibitors. A number of compounds that synthesized in this study have showed potent and selective inhibitory activity against butyrylcholinesterase (BuChE), which is comparable to the activity of some previously reported quinazoline derivatives^25,32–34^.

The structure–activity relationship investigation of the synthesized compounds indicated that the position and the nature of the substitutions on the phenyl rings significantly affect the eelAChE and eqBuChE inhibitory activities. Generally, the synthesized compounds showed higher potency and selectivity to inhibit eqBuChE compared to eelAChE. From the biological results, it was found that the introduction of one methyl group (**6f** and **6j**) or two methoxy groups (**6h**) on the phenyl ring improves the inhibitory activity against both eelAChE and eqBuChE. While the presence of electron donating substitutions such as OH and NH_2_ (**6b** and **6c**) and electron withdrawing substitutions such as Cl, Br, and CF_3_ (**6a**, **6e**, **6d**, and **6i**) at the 2-position of the phenyl rings significantly decrease the eqBuChE inhibitory activity of the synthetic compounds. Comparing the inhibitory activity of compounds **6g** and **6i**, each containing one methoxy group, to compound **6h** with two methoxy groups, showed that the presence of two methoxy groups on the phenyl ring is much more effective for eqBuChE inhibitory activity compared to one methoxy group. The eqBuChE inhibition can be ranked as follows: 4-Me > 2-OH-5-Me > 2,4-OMe > 2-NH_2_ > 2-OH > 3-Br, 3-CF_3_. Further investigation could help identify the most effective substitutions and positions that optimize the inhibitory capacity of these compounds.

### Kinetic studies of enzyme inhibition

**Figure 3:**
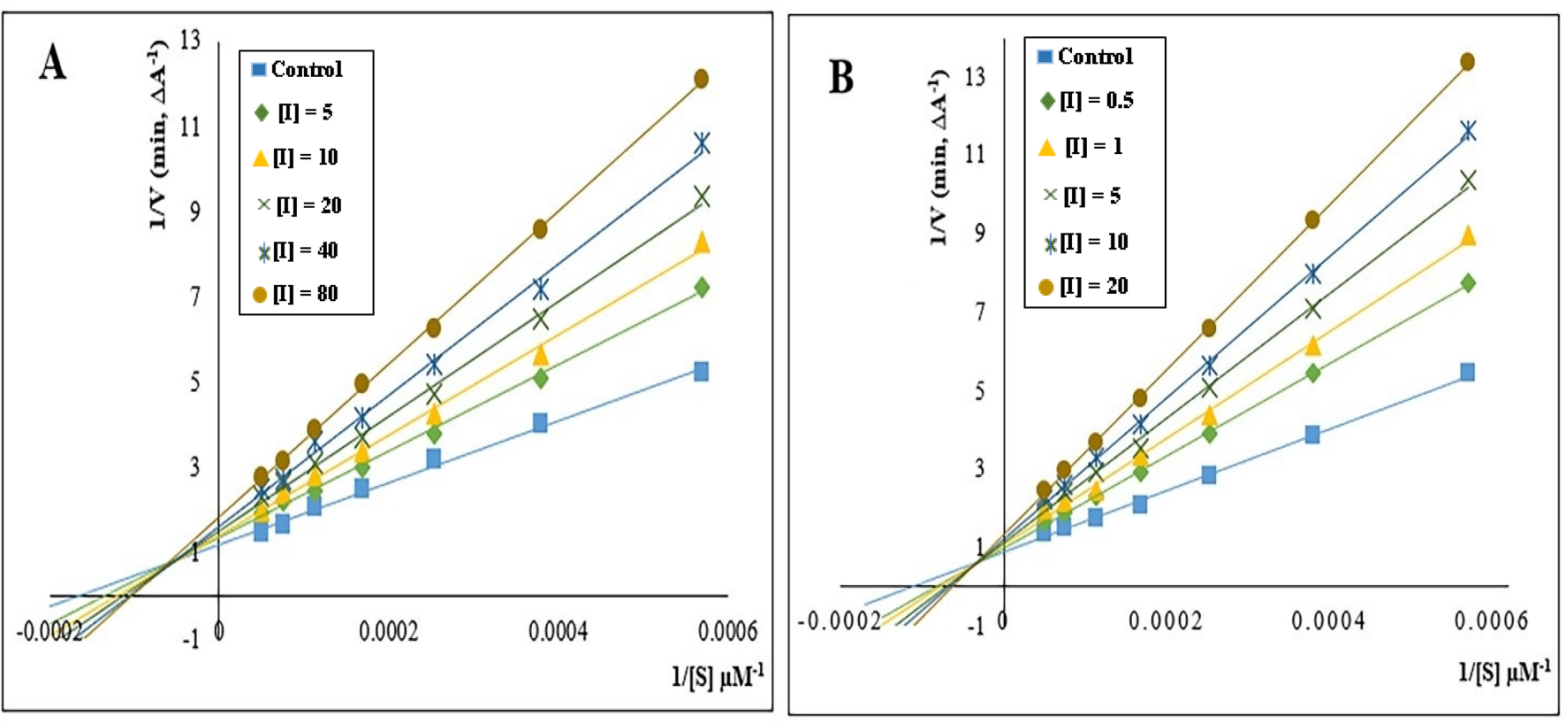
(**A**) Lineweaver–Burk plot for the inhibition of eelAChE by compound **6h**; (**B**) Lineweaver–Burk plot for the inhibition of eqBuChE by compound **6f**.

Kinetic study was performed to investigate the inhibition mechanism of eelAChE and eqBuChE using graphical analysis of the reciprocal Lineweaver–Burk plot. Compounds **6h** and **6f**, as the most potent inhibitors, were selected for eelAChE and eqBuChE, respectively. The kinetic studies were performed in the absence (control) and presence of different concentrations of compound **6h** (5, 10, 20, 40, and 80 μM) for eelAChE and different concentrations of compound **6f** (0.5, 1, 5, 10, and 20 μM) for eqBuChE. The control wells contained no concentration of **6h** and **6f** as inhibitors. As demonstrated in Fig. 3, with increasing concentrations of inhibitors (**6h** and **6f**), the values of V_max_ decreased, and the values of K_m_ increased. These patterns indicated a mixed-type inhibition for both enzymes, which revealed that compounds **6h** and **6f** might be able to bind to the catalytic active site (CAS) and peripheral anionic site (PAS) of eelAChE and eqBuChE.

### Antioxidant activity

The DPPH method presents several advantages, such as low cost, ease of performing experiments, high reproducibility, applicability at room temperature, and automation possibilities. In contrast to other traditional antioxidant assays that require additional treatments of sample and reagents, such as high temperatures and/or oxygen supply, the DPPH method requires mild experimental conditions^35,36^. These features have established it as a convenient and effective option for researchers in the evaluation of antioxidant properties. In this study, the antioxidant activities of the synthesized compounds **6a-j** were assessed using the DPPH assay, and the obtained results are represented as EC_50_ values in Table 2. In this assay, quercetin was used as positive control to compare the antioxidant capacity of the tested compounds. Among all the synthesized compounds, **6b**, **6c**, and **6j**, which bear OH and NH_2_ substitutions at the *ortho* position of the phenyl rings, displayed the best scavenging activity, with EC_50_ values of 26.1, 28.4, and 23.2 µM, respectively. The antioxidant results indicated that the presence of OH and NH_2_ substitutions on the phenyl rings improves the antioxidant capacity of the synthesized compound.

**Table 2:** The antioxidant activities of the synthesized compounds **6a**–**j** using DPPH assay.


Compounds	R	EC_50_ (µM)
6a	2-Br	> 400
6b	2-OH	26.1
6c	2-NH_2_	28.4
6d	3-Br	> 400
6e	3-CF_3_	> 400
6f	4-Me	> 400
6g	4-OMe	> 400
6h	2,4-di-OMe	> 400
6i	5-Cl-2-OMe	> 400
6j	2-OH-5-Me	23.2
Quercetin	–	8.9

Among compounds **6f**, **6h**, and **6j**, which were the most active BuChE inhibitors, compounds **6f** and **6h** showed EC_50_ values of greater than 400 µM, while compound **6j** with 2-hydroxy-5-methyl (2-OH-5-Me) substitutions displayed good antioxidant activity with EC_50_ = 23.2 µM.

### Molecular docking study

**Figure 4:**
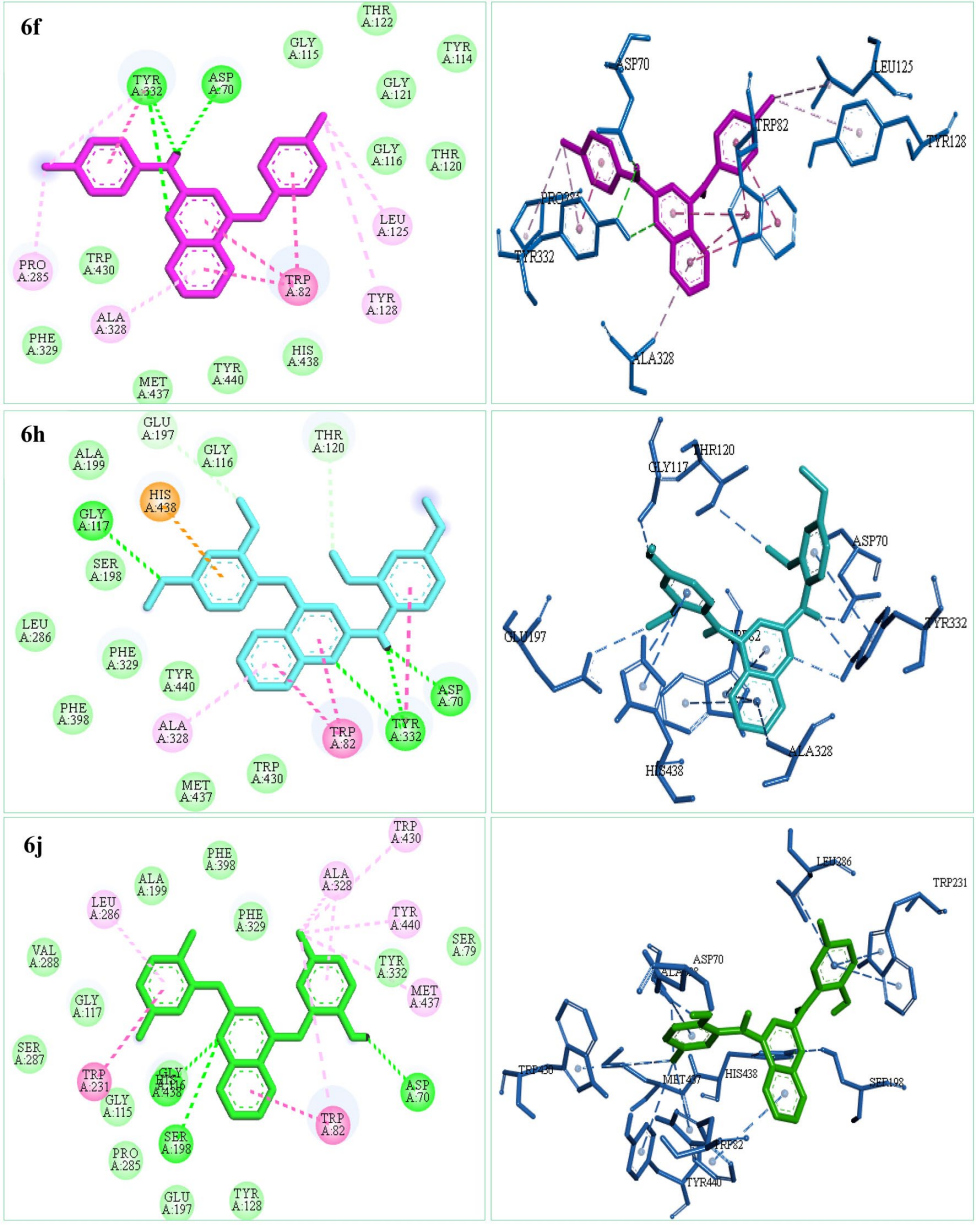
2D and 3D interactions of compounds **6f**, **6h**, and **6j** in the active site of BuChE (1P0I). (Green: Van der Waals, light green: carbon hydrogen bond, dark green: hydrogen bond, dark pink: π−π, light pink: alkyl & π-alkyl, purple: π-sigma, orange: π-sulfur).

To explore the possible interactions of 2,4-disubstituted quinazoline derivatives (**6a-j**) with the BuChE active site, molecular docking studies were carried out using a BuChE crystal structure retrieved from the RCSB Protein Data Bank with PDB ID: 1P0I. The molecular docking results of compounds **6f**, **6h**, and **6j** with the best in vitro eqBuChE inhibitory activities displayed in Fig. 4. In general, compounds **6f**, **6h**, and **6j** showed a similar binding pattern with binding free energies of − 11.2, − 9.9 and − 10.6 kcal/mol, respectively.

According to the studies, the catalytic active site (CAS) of BuChE consists of a choline binding pocket (Trp82), a peripheral anion site (Asp70), catalytic residues (Ser198, His438, and Glu325), and an acyl-binding pocket (Val288, Leu286, and Trp231). Among these residues, Ser198 and Trp82 are the key residues for the enzyme activity^20^.

As depicted in Fig. 4, compounds **6f**, **6h**, and **6j** bind to the BuChE active site by forming strong hydrogen bonds with Asp70, Tyr332, Gly116, Gly117, Glu197, Ser198, and His438 residues. In the case of compound **6f**, π−π stacking interactions are observed between the quinazoline moiety and Trp82 as an important amino acid in the catalytic active site of BuChE, as well as between the phenyl rings and Trp82 and Tyr332 residues. In compound **6h**, the quinazoline ring and one of the phenyl rings interact with Trp82 and Tyr332 residues via π−π-stacking interactions, while another phenyl ring forms a π-cation interaction with His438. In the case of compound **6j**, the quinazoline ring and one of the phenyl rings are involved in π−π stacking interactions with Trp82 and Trp231 residues. As shown in Fig. 4, these compounds also form alkyl and van der Waals interactions with various residues, including Ala328, Pro285, Leu125, Tyr128, Leu286, Gln119, Phe398, etc.

These findings demonstrate that the most active compounds **6f**, **6h**, and **6j** are well fixed in the active site of BuChE and interact with the critical residues. The docking results are consistent with the biological findings and offer a logical view for the design more effective and novel BuChE inhibitors.

### MD simulation

**Figure 5:**
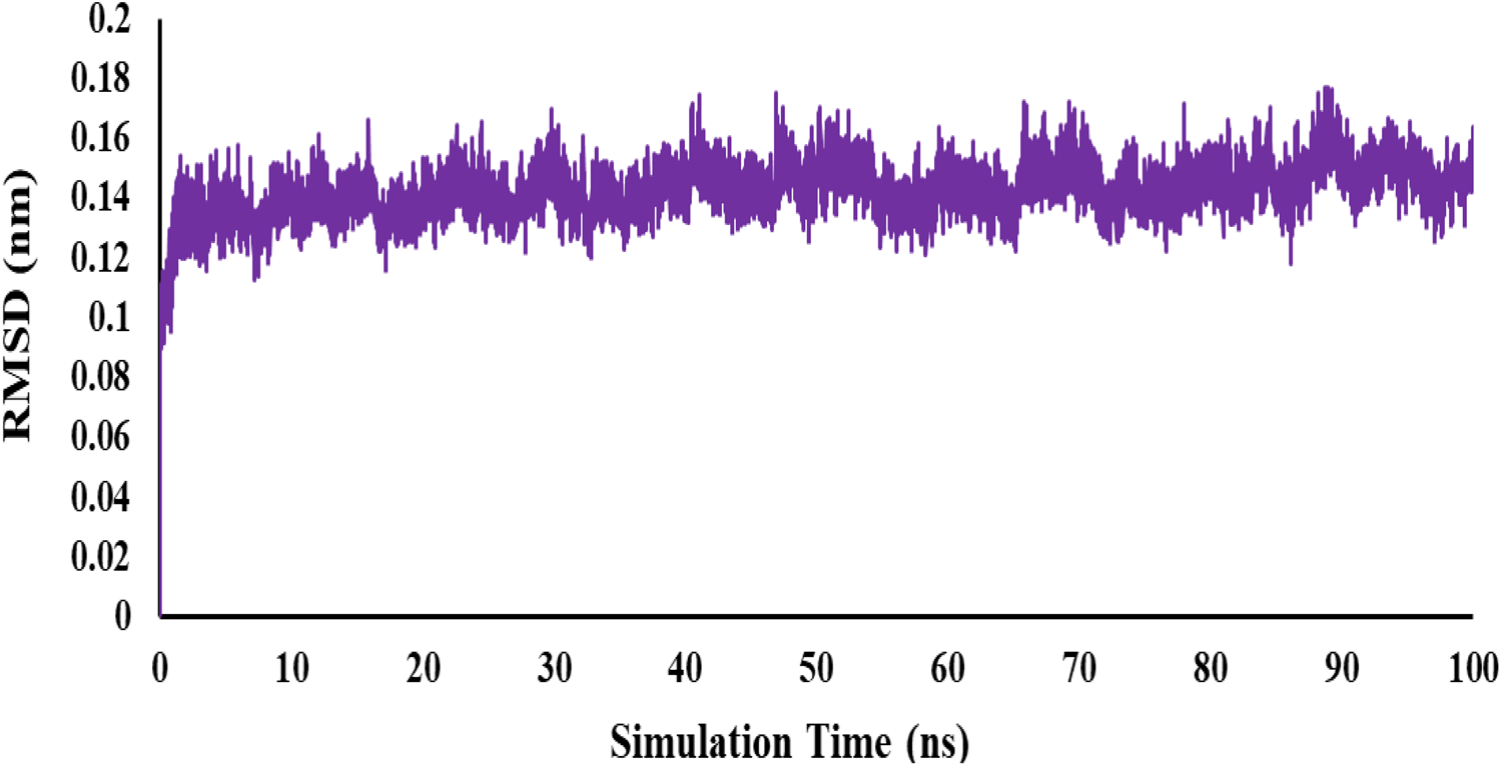
The RMSD analysis of a complex involving the compound **6f** interacting with the hBuChE indicated that **6f** stabilized inside the hBuChE after 10 ns of simulation.

Molecular dynamics (MD) simulation is a valuable technique for examining the structures and interactions of biological molecules^37^. In this investigation, the main focus was on the interaction between the atoms of the active molecule **6f** during a 100-ns MD simulation. The main goal of this simulation was to evaluate the structural stability of the enzyme. The Root-Mean-Square deviation (RMSD) values, which measure the amount of atomic positional variation in different frames relative to a reference frame, were calculated to quantify this stability. The backbone atoms of each frame were compared to those in the simulation's first frame in order to perform alignment. Compound **6f** achieved equilibrium phases at about 10 ns, based on the consistent RMSD profile (Fig. 5). This suggests that the ligand becomes anchored in the active site of human butyrylcholinesterase (hBuChE). The RMSD profile's consistent pattern indicates that the ligand was stable throughout the simulation. Furthermore, with respect to the 1P0I protein (which is taken to be a reference structure), the RMSD values for compound **6f** remained noticeably consistent. This suggests that throughout the simulation, the ligand maintained a constant conformation and contact with the hBuChE receptor.

**Figure 6:**
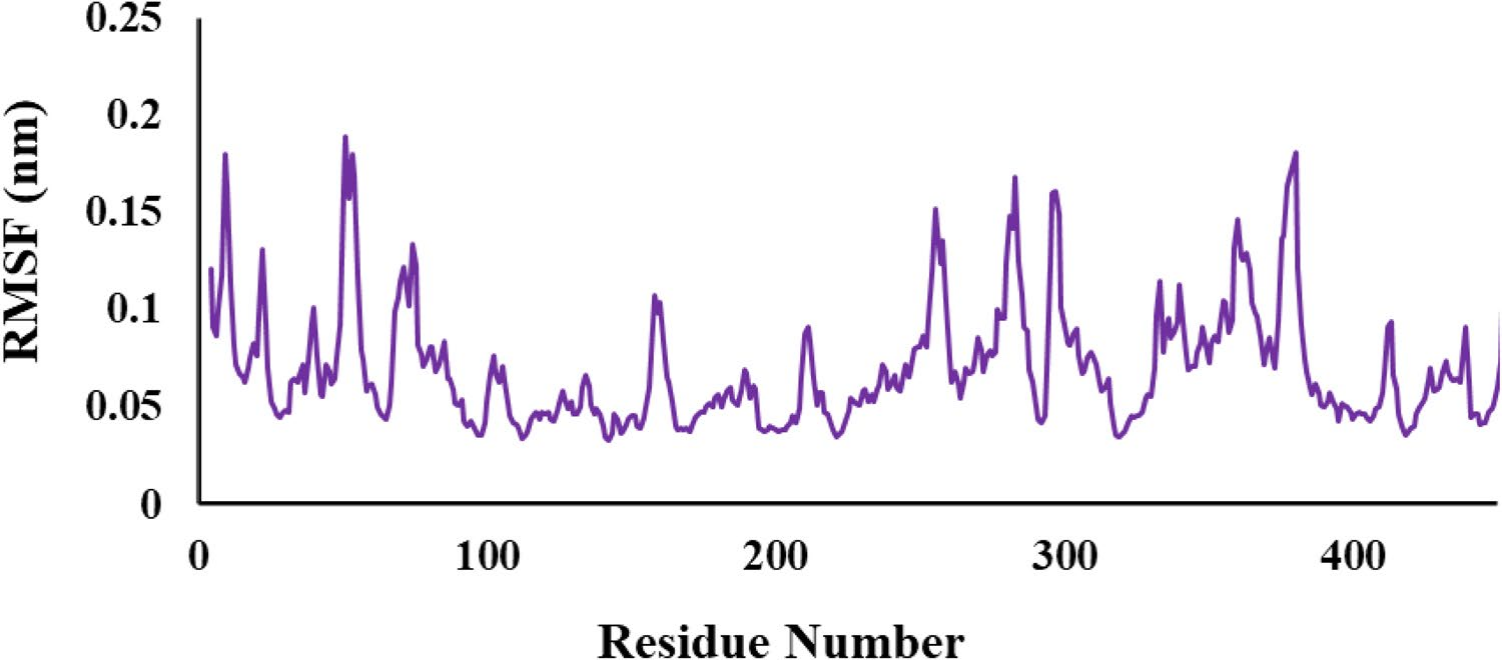
The assessment of RMSF value was performed on the complex involving the compound **6f**.

To study the flexibility of the protein when bound with a ligand, we thoroughly examined the root mean square fluctuation (RMSF) of the protein backbone during the simulation time (Fig. 6). The results of the investigation showed that the distribution of RMSF values for the ligand **6f** complex suggested that the general flexibility of the protein is not considerably changed by the binding of the ligand. Moreover, none of the protein kinase's active site residues had an RMSF value larger than 0.18 nm, indicating that ligand interaction did not cause any appreciable alterations in these residues' flexibility. As a result, during the equilibrium time range, the active site remained rather steady.

**Figure 7:**
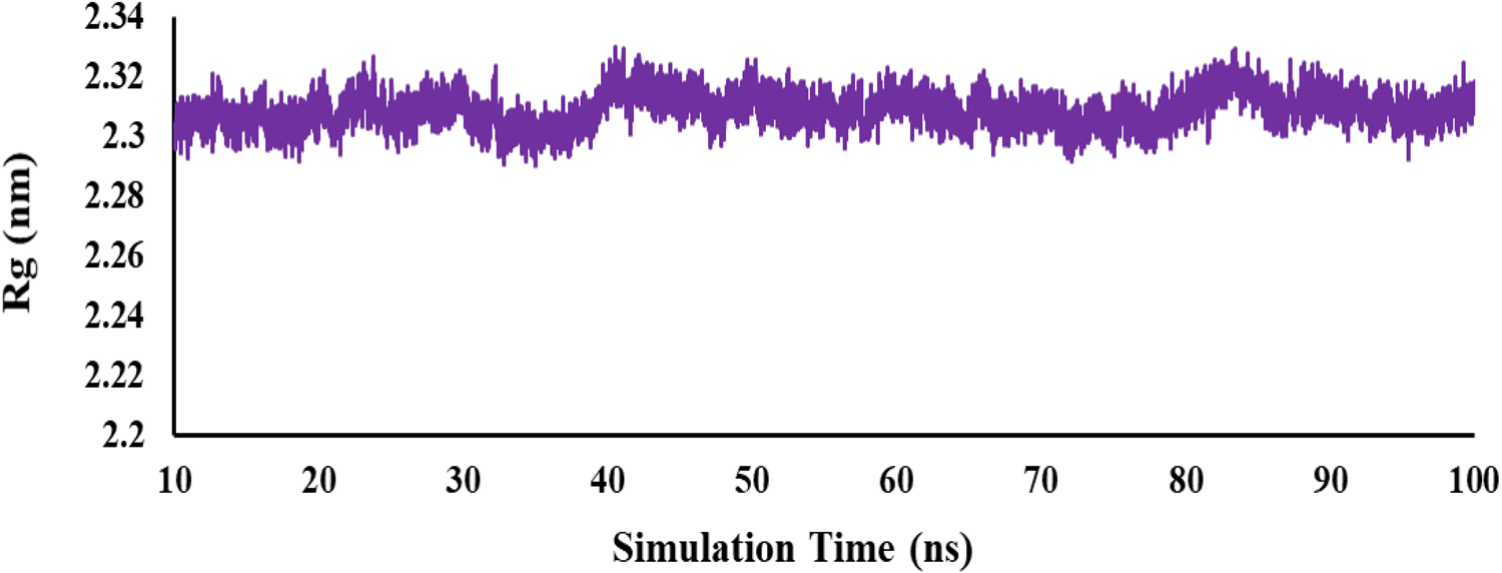
The radius of gyration (Rg) plot of compound **6f** in the equilibrium time range.

The evaluation of the radius of gyration (Rg) parameter indicates changes in compactness within a ligand-protein complex. The analysis suggests that the protein became more compact over the simulation duration, implying some compression occurred. Interestingly, the Rg plot displays that compound **6f** complex reaches an Rg value plateau of 2.3 nm. This suggests that the interaction with compound **6f** maintains the stability and compactness of the protein (as depicted in Fig. 7).

**Figure 8:**
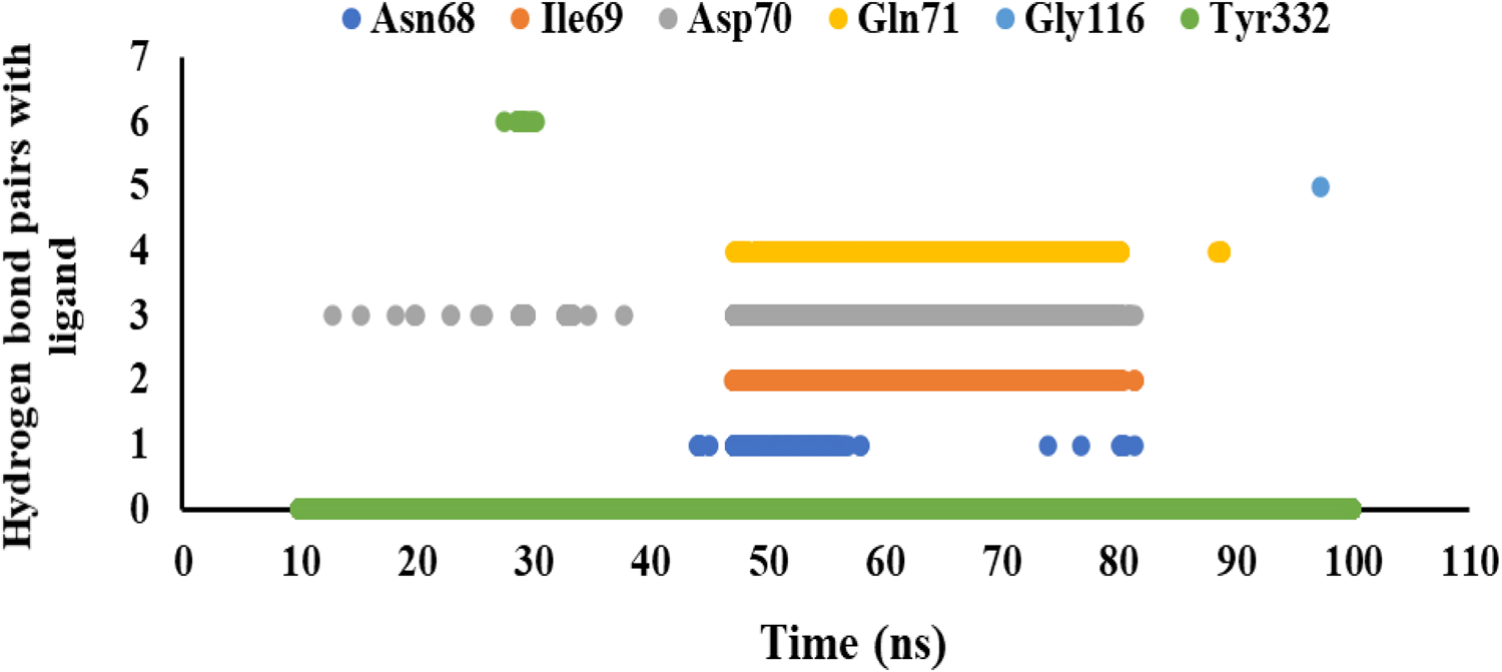
The stability of hydrogen bond interactions between **6f** and promising residues inside the active site of hBuChE in the equilibrium time range.

We monitored the lifetime of hydrogen bond interactions between the ligand and amino acid residues within the active site of hBuChE during the equilibrium time range. The key residues for hydrogen bond interactions were Asn68, Ile69, Asp70, Gln71, Gly116, and Tyr332, while the stability of residues was different per compound during the equilibrium time range. As depicted in Fig. 8, stable hydrogen bond interactions (more than 34%) were observed with Asp70 and Gln71 residues for compound **6f**.

**Figure 9:**
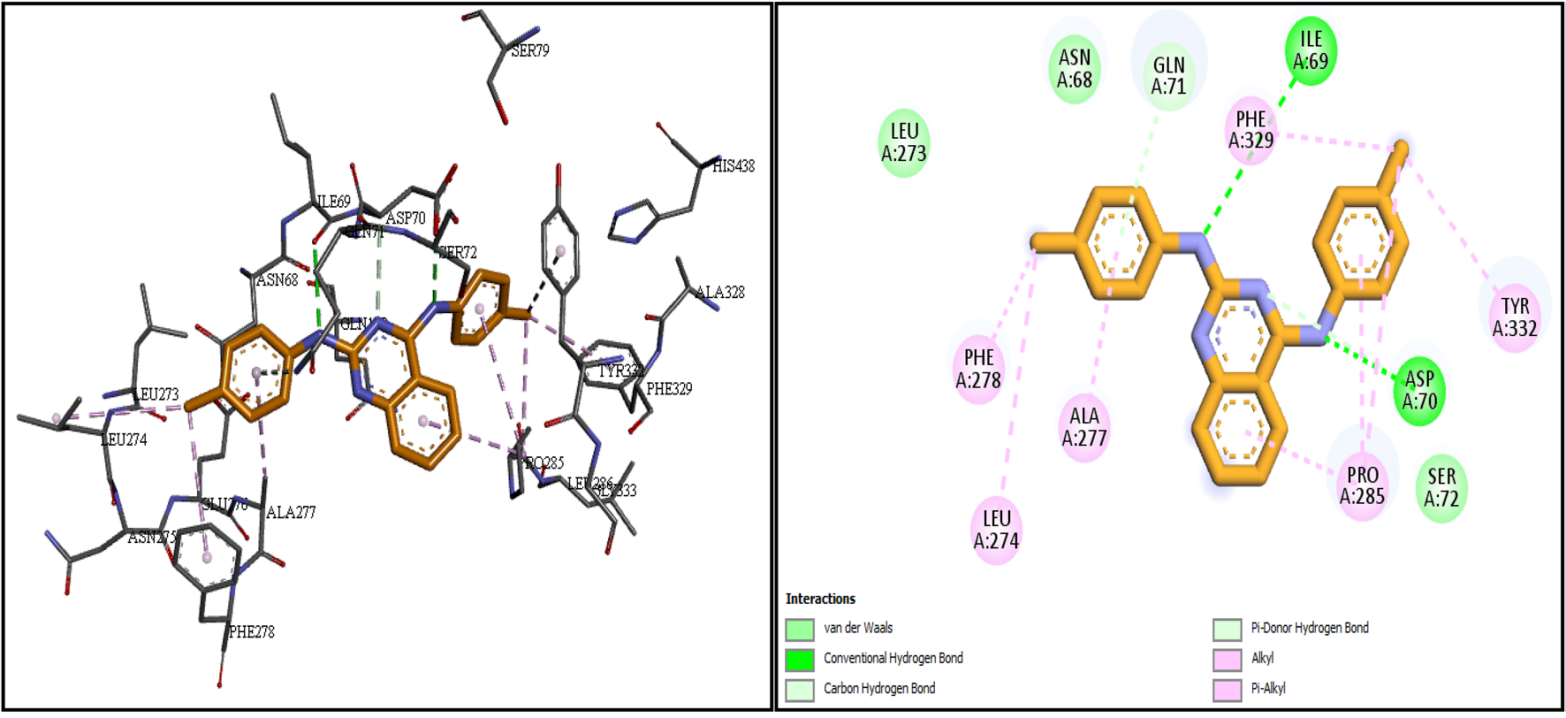
Molecular dynamics (MD) simulation analysis of **6f** with hBuChE.

Analysis of the interaction of compound **6f** with the hBuChE revealed two critical hydrogen bonds from the NH group with Ile69 and Asp70 residues. Additionally, two important carbon hydrogen interactions were observed between the N atom of the quinazoline ring and the phenyl ring with Asp70 and Gln71, respectively. Moreover, compound **6f** made π-alkyl and alkyl interactions with Lue274, Ala277, Phe278, Pro285, Phe329, and Tyr332. Furthermore, van der Waals interactions with Ser72, Asn68, and Leu273 residues were detected (Fig. 9). Finally, the result demonstrate that compound **6f** makes good interactions with hydrophobic pocket of the hBuChE receptor and is completely consistent with the docking studies.

## Material and methods

### Chemistry

All commercially available reagents and solvents were purchased from Sigma-Aldrich and Merck Chemical Company and used without further purification. ^1^H-NMR and ^13^C-NMR spectra were recorded in DMSO-d6 on Bruker 400 MHz instrument and the chemical shifts were reported in terms of parts per million (ppm, δ) with TMS as the internal reference and coupling constants were reported in Hertz (Hz). Proton coupling patterns were described as singlet (s), doublet (d), triplet (t), quartet (q), multiplet (m), and broad (br). Mass spectra were obtained using Agilent Technologies (HP) MS with an API-ES ionization source. Melting points were measured in capillary tubes with an electrothermal 9200 instrument.

### Synthesis of quinazoline-2,4-*dione* (3)

Quinazoline-2,4-dione (**3**) was synthesized through the cyclization of commercially available anthranilic acid with urea at 190 °C. For this purpose, 0.15 mol of urea was melted at 190 °C, and 0.1 mol of anthranilic acid was added slowly to it. The reaction mixture was stirred at this temperature for 24 h. After the completion of reaction, the reaction mixture was cooled to room temperature, and the resulting precipitate was washed with distilled water and purified by recrystallization with ethyl acetate to afford quinazoline-2,4-dione with 40% yield.

### Synthesis of 2,4-dichloroquinazoline (4)

A mixture of quinazoline-2,4-dione (**3**) (2.0 g), phosphoryl chloride (POCl_3_) (6.0 mL) and *N*,*N*-diethylaniline (0.6 mL) was refluxed at 115 °C for 16 h. After the completion of reaction, the reaction mixture was cooled to room temperature and a mixture of distilled water and ice was added to it. The resulting precipitate was then isolated, washed with distilled water and purified by recrystallization with n-hexane to obtain the expected 2,4-dichloroquinazoline with 44.8% yield.

^1^H-NMR (DMSO-d_6_, 400 MHz) δ: 8.07 (dd*, J* = 2.4, 12.4 Hz, 1H), 7.82 (t,* J* = 11.2 Hz, 1H), 7.61–7.50 (m, 2H).

### General procedure for the preparation of compounds 6a-j

A mixture of 2,4-dichloroquinazoline (**4**) (1 mmol) and various aniline derivatives (3 mmol) in ethanol (20 mL) was refluxed for 24 h. After the completion of reaction, the reaction mixture was cooled to room temperature. In the following, the resulting precipitate was isolated and washed with n-hexane to obtain the target quinazoline derivatives **6a**–**j** with high yields.

#### N^2^, N^4^-bis(2-bromophenyl) quinazoline-2,4-diamine (6a)

White solid; yield: 63.1%; mp: 250–252 °C; IR (KBr) ν (cm^−1^): 3322 (N–H, stretching), 3078 (C–H, aromatic), 1534–1495 (C=C, aromatic), 1620, 1593 (C=N, aromatic), 743 (C–H, bending, aromatic); ^1^H-NMR (400 MHz, DMSO-d_6_) δ (ppm): 11.64 (s, 1H, NH),10.00 (s, 1H, NH), 8.71 (d, *J* = 8.0 Hz, 1H), 7.97 (t, *J* = 7.6 Hz, 1H), 7.78 (d, *J* = 8.0 Hz, 1H), 7.68 (d, *J* = 8.4 Hz, 1H), 7.64–7.59 (m, 2H), 7.56 (d, *J* = 8.8 Hz, 2H), 7.49 (t, *J* = 7.6 Hz, 1H), 7.33 (t, *J* = 7.2 Hz, 1H), 7.17–7.10 (m, 2H). ^13^C-NMR (100 MHz, DMSO) δ (ppm): 165.7, 156.9, 144.4, 141.5, 140.8, 139.4, 138.1, 137.9, 135.2, 134.7, 133.6, 132.9, 132.7, 131.6, 130.7, 130.6, 130.2, 126.8, 122.8, 115.2. MS: m/z (%): 474.4 [M + 4] (0.10), 472.4 [M + 2] (0.07), 470.1 [M^+^] (0.12), 391.2 (55.60), 309.3 (100), 219.2 (12.92), 155.2 (56.23), 90.1(15.28).

#### 2,2′-(quinazoline-2,4-diylbis(azanediyl))diphenol (6b)

White solid; yield: 66.3%; mp: 260–266 °C, ^1^H-NMR (400 MHz, DMSO-d_6_) δ (ppm): 11.03 (s, 1H, NH), 9.67 (s, 1H, NH), 9.33 (s, 1H, OH), 8.28 (d, *J* = 8.4 Hz, 1H), 8.20 (s, 1H, OH), 7.79 (d, *J* = 8.0 Hz, 1H), 7.68 (t, *J* = 7.2 Hz, 1H), 7.61 (d, *J* = 7.6 Hz, 1H), 7.43 (d, *J* = 8.0 Hz, 1H), 7.29 (t, *J* = 7.2 Hz, 1H), 7.13 (t, *J* = 7.2 Hz, 1H), 6.98 (d, *J* = 8.0 Hz, 1H), 6.92–6.84 (m, 3H), 6.72–6.70 (m, 1H). ^13^C-NMR (100 MHz, DMSO-d_6_) δ (ppm): 159.5, 156.5, 151.5, 150.1, 147.2, 133.2, 128.7, 127.2, 126.5, 126.5, 125.8, 124.4, 123.2, 122.7, 122.2, 120.8, 119.0, 116.6, 116.0, 111.4. MS: m/z (%): 344.2 (97.77), 327.2 (100), 310.1 (23.77), 236.1 (94.31), 211.1 (38.66), 210.1 (85.27), 181.1 (13.57), 135.0 (12.67), 109.0 (29.73), 65.1 (30.51).

#### N^1^, N^1′^-(quinazoline-2,4-diyl)bis(benzene-1,2-diamine) (6c)

White solid; yield: 71.5%; mp: 220–223 °C; ^1^H-NMR (400 MHz, DMSO-d_6_) δ (ppm): 11.6 (s, 1H, NH), 9.99 (s, 1H, NH), 8.70 (d, *J* = 8.0 Hz, 1H), 7.96 (t, *J* = 7.6 Hz, 1H), 7.78 (d, *J* = 7.2 Hz, 1H), 7.68 (d, *J* = 8.4 Hz, 1H), 7.64–7.63 (m, 2H), 7.57–7.55 (m, 2H), 7.49 (t, *J* = 7.2 Hz, 1H), 7.33 (t,* J* = 7.6 Hz, 1H), 7.19–7.10 (m, 2H). ^13^C-NMR (100 MHz, DMSO) δ (ppm): 160.4, 151.7, 139.3, 136.1, 135.5, 134.2, 132.8, 132.7, 129.9, 129.4, 128.4, 127.8, 127.5, 126.5, 125.4, 124.7, 121.5, 117.6, 109.9. MS: m/z (%): 342.1 (44.20), 324.1 (25.97), 235.1 (80.84), 209.1 (100), 208.1 (37.50), 181.0 (16.99), 133.0 (41.12), 108.1 (62.42), 80.0 (41.57), 65.1 (35.59), 52.0 (17.61).

#### N^2^,N^4^-bis(3-bromophenyl)quinazoline-2,4-diamine (6d)

White solid; yield: 93.5%; mp: 310–313 °C; IR (KBr) ν (cm^−1^): 3256, 3188 (N–H, stretching), 3052 (C–H, aromatic), 1568–1474 (C=C, aromatic), 1606,1595 (C=N, aromatic), 766 (C–H, bending, aromatic); ^1^H-NMR (400 MHz, DMSO-d_6_) δ (ppm): 11.26 (s, 1H, NH),10.73 (s, 1H, NH), 8.69 (d, *J* = 8.0 Hz, 1H), 7.95–7.91 (m, 2H), 7.73 (s, 2H), 7.66 (d, *J* = 8.4 Hz, 1H), 7.58 (t, J = 7.6 Hz, 1H), 7.49 (t,* J* = 8.4 Hz, 2H), 7.42 (t, *J* = 7.2 Hz, 1H), 7.37 (d, *J* = 8.0 Hz, 1H), 7.32 (t, *J* = 7.6 Hz, 1H).^13^C-NMR (100 MHz, DMSO-d_6_) δ (ppm): 159.6, 151.6, 138.5, 138.4, 135.9, 130.7, 130.6, 129.0, 127.5, 127.1, 125.0, 124.8, 124.4, 123.5, 121.6, 121.3, 120.9, 117.9, 110.6. MS: m/z (%): 474.1 [M + 4] (0.77), 472.1 [M + 2] (34.79), 470.1 [M^+^] (67.99), 389.2 (16.98), 309.3 (20.27), 273.1 (45.18), 219.2 (48.17), 154.8 (100), 129.1 (8.60), 102.2 (16.91), 76.2 (39.52).

#### N^2^, N^4^-bis(3-(trifluoromethyl)phenyl)quinazoline-2,4-diamine (6e)

White solid; yield: 75.7%; mp: 288–290 °C; IR (KBr) ν (cm^−1^): 3205 (N–H, stretching), 3044 (C–H, aromatic), 1545–1493 (C=C, aromatic), 1639, 1584 (C=N, aromatic), 756 (C–H, bending, aromatic), 1019 (C–F, stretching); ^1^H-NMR (400 MHz, DMSO-d_6_) δ (ppm): 11.44 (s, 1H, NH),10.86 (s, 1H, NH), 8.75 (d, *J* = 8.0 Hz, 1H), 8.01 (br, 2H), 7.95 (t, *J* = 8.0 Hz, 1H), 7.83 (s, 1H), 7.73 (d, *J* = 7.6 Hz, 1H), 7.70 (d, *J* = 8.4 Hz, 1H), 7.62–7.57 (m, 3H), 7.55–7.49 (m, 2H). ^13^C-NMR (100 MHz, DMSO-d_6_) δ (ppm): 160.3, 152.1, 140.0, 138.1, 137.9, 136.4, 130.4 (C–F), 130.3 (C–F), 130.2 (C–F), 130.1 (C–F), 129.8, 129.7, 128.9, 126.4, 126.1 (C–F), 126.0 (C–F), 125.6 (C–F), 125.5 (C–F), 123.2, 123.1, 122.5, 122.4, 121.7, 121.6, 118.9, 119.0, 118.2, 111.1. MS: m/z (%): 447.3 [M^+^] (0.07), 379.3 (6.33), 288.1 (13.78), 263.2 (81.17), 214.2 (14.17), 179.2 (13.93), 145.1 (39.45), 125.1 (12.93), 95.1 (16.33).

#### N^2^, N^4^-di-p-tolylquinazoline-2,4-diamine (6f)

White solid; yield: 87.9%; mp: 290–295 °C; ^1^H-NMR (400 MHz, DMSO-d_6_) δ (ppm): 11.15 (s, 1H, NH), 10.55 (s, 1H, NH), 8.70 (d, *J* = 8.0 Hz, 1H), 7.88 (t, *J* = 7.6 Hz, 1H), 7.60–7.50 (m, 4H), 7.34 (d,* J* = 8.4 Hz, 2H), 7.24 (d, *J* = 8.0 Hz, 2H), 7.12 (d, *J* = 8.0 Hz, 2H), 2.36 (s, 3H, methyl), 2.30 (s, 3H, methyl). ^13^C-NMR (100 MHz, DMSO-d_6_) δ (ppm): 159.2, 151.3, 139.0, 135.6, 135.5, 134.3, 134.2, 134.1, 133.9, 129.2, 128.9, 124.8, 124.6, 122.0, 117.1, 110.3, 20.6, 20.4. MS: m/z (%): 340.2 (100), 249.1 (7.42), 209.1 (50.38), 155.0 (11.31), 106.0 (21.93), 91.0 (35.76), 77.0 (19.42), 65.0 (26.91), 36.0 (11.09).

#### N^2^, N^4^-bis(4-methoxyphenyl)quinazoline-2,4-diamine (6g)

White solid; yield: 83.5%; mp: 287–290 °C; ^1^H-NMR (400 MHz, DMSO-d_6_) δ (ppm): 11.13 (s, 1H, NH), 10.43 (s, 1H, NH), 8.70 (d, *J* = 8.0 Hz, 1H), 7.86 (t, *J* = 7.6 Hz, 1H), 7.58 (d, *J* = 6.8 Hz, 3H), 7.50 (t, *J* = 7.2 Hz, 1H), 7.35 (d, *J* = 8.0 Hz, 2H), 6.97 (d, *J* = 8.0 Hz, 2H), 6.90 (d, *J* = 6.4 Hz, 2H), 3.79 (s, 3H, methoxy), 3.76 (s, 3H, methoxy). ^13^C-NMR (100 MHz, DMSO-d_6_) δ (ppm): 158.9, 157.3, 151.6, 139.1, 135.4, 129.7, 126.0, 124.7, 124.5, 117.2, 113.9, 113.6, 110.3, 55.3, 55.2. MS: m/z (%): 372.1 (56.68), 358.1(9.62), 357.1 (36.48), 293.2 (93.48), 212.1 (44.98), 211.1 (100), 183.1 (45.24), 182.1 (41.52), 171.0 (41.37), 143.1 (63.97), 115.0 (36.33), 96.1 (82.70), 55.1 (33.74).

#### N^2^, N^4^-bis(2,4-dimethoxyphenyl)quinazoline-2,4-diamine (6h)

White solid; yield: 86.7%; mp: 257–259 °C; ^1^H-NMR (400 MHz, DMSO-d_6_) δ (ppm): 10.78 (s, 1H, NH), 9.38 (s, 1H, NH), 8.56 (d, *J* = 7.6 Hz, 1H), 7.86 (t, *J* = 7.2 Hz, 1H), 7.57–7.47 (m, 3H), 7.32 (d, *J* = 8.4 Hz, 1H), 6.76 (s, 1H), 6.63 (d, *J* = 7.6 Hz, 2H), 6.29 (s, 1H), 3.84 (s, 3H, methoxy), 3.79 (s, 3H, methoxy), 3.76 (s, 6H, methoxy). ^13^C-NMR (100 MHz, DMSO-d_6_) δ (ppm): 160.2, 159.6, 155.0, 135.4, 128.7, 124.5, 118.1, 117.7, 109.99, 104.7, 104.3, 99.1, 98.7, 55.8, 55.7, 55.5, 55.3. MS: m/z (%): 432.2 (73.90), 402.2 (61.50), 401.3 (100), 371.1 (11.65), 343.1 (9.44), 250.1 (13.48), 216.1 (20.80), 179.1 (6.65), 138.0 (16.91), 110.0 (6.53), 79.0 (11.59), 52.0 (4.49).

#### N^2^, N^4^-bis(5-chloro-2-methoxyphenyl)quinazoline-2,4-diamine (6i)

White solid; yield: 70.2%; mp: 249–252 °C; IR (KBr) ν (cm^−1^): 3449, 3189 (N–H, stretching), 3052 (C–H, aromatic), 2934 (C–H, aliphatic), 1549–1495 (C=C, aromatic), 1625, 1604 (C=N, aromatic), 1028 (C–O, stretching), 754 (C–H, bending, aromatic); ^1^H-NMR (400 MHz, DMSO-d_6_) δ (ppm): 11.28 (s, 1H, NH), 9.71 (s, 1H, NH), 8.63 (d, *J* = 8.4 Hz, 1H), 7.96 (t, *J* = 7.6 Hz, 1H), 7.66 (s, 1H), 7.62–7.57 (m, 2H), 7.54–7.53 (m, 1H), 7.49–7.46 (dd, *J* = 2.4, 7.2 Hz, 1H), 7.27 (d, *J* = 8.8 Hz, 1H), 7.13–7.07 (m, 2H), 3.85 (s, 3H, methoxy), 3.79 (s, 3H, methoxy).^13^C-NMR (100 MHz, DMSO-d_6_) δ (ppm): 160.6, 152.9, 150.9, 139.0, 136.2, 128.9, 127.6, 126.7, 126.1, 125.3, 124.9, 124.1, 123.9, 117.3, 114.1, 112.6, 109.8, 56.3, 56.0. MS: m/z (%): 442.4 [M + 2] (1.29), 440.2 [M^+^] (1.37), 409.2 (100), 377.1 (15.99), 254.1 (8.29), 206.2 (8.22), 165.2 (12.23), 142.1 (16.50), 102.1 (15.16), 78.1 (23.32).

#### 2'-(quinazoline-2,4-diylbis(azanediyl))bis(4-methylphenol) (6j)

White solid; yield: 67.9%; mp: 337–339 °C; IR (KBr) ν (cm^−1^): 3361 (N–H, stretching), 3256 (O–H, stretching), 3086 (C-H, aromatic), 2944 (C–H, aliphatic), 1554–1498 (C=C, aromatic), 1622, 1585 (C=N, aromatic), 749 (C–H, bending, aromatic); ^1^H-NMR (400 MHz, DMSO-d_6_) δ (ppm): 10.99 (s, 1H, NH), 10.19 (s, 1H, NH), 9.70 (s, 1H), 9.31 (s, 1H, OH), 8.61 (d, *J* = 8.0 Hz, 1H), 7.90 (t, *J* = 7.6 Hz, 1H), 7.58–7.47 (m, 3H), 7.16 (s, 1H), 7.05 (d, *J* = 8.4 Hz, 1H), 6.97 (d, *J* = 8.4 Hz, 1H), 6.80 (d, *J* = 7.6 Hz, 1H), 6.71 (s, 1H, OH), 2.22 (s, 3H, methyl), 1.95 (s, 3H, methyl). ^13^C-NMR (100 MHz, DMSO-d_6_) δ (ppm): 160.4, 150.1, 139.1, 135.6, 129.1, 128.2, 127.7, 127.6, 124.8, 124.7, 123.4, 117.1, 116.5, 109.9, 20.3, 20.0. MS: m/z (%): 372.1 [M^+^] (0.18), 355.3 (88.75), 250.2 (69.06), 224.1 (54.41), 206.1 (3.37), 186.1 (11.69), 165.2 (13.27), 144.1 (10.27), 123.1 (29.58), 102.1 (13.76), 77.1 (76.22), 43.1 (100).

### Biological activity

#### AChE and BuChE inhibition assay

The inhibitory activities of the synthesized compounds towards AChE and BuChE enzymes were assessed based on the Ellman’s method. Acetylcholinesterase from the electric eel (eelAChE), butyrylcholinesterase from equine serum (eqBuChE), 5,5′-dithiobis-(2-nitrobenzoic acid) (DTNB), acetylthiocholine iodide (ATCI), butyrylthiocholine iodide (BTCI) and donepezil as control drug were obtained from Sigma Aldrich. Each well contained 200 µL of phosphate buffer (0.1 M, pH = 7.4), 20 µL of DTNB (0.004 M), 20 µL of eqBuChE or eelAChE solutions and 10 µL of the desired concentrations of the tested compounds (3–50 µM). The plates were incubated for 15 min at room temperature. Subsequently, 20 µL of acetylthiocholine iodide (0.006 M) or butyrylthiocholine iodide (0.006 M) was added to the incubated mixture and the change in the absorbance were recorded at 412 nm using a 96-well plate reader (Synergy HTX Multi-Mode Reader-BioTek). The control wells were prepared under the same conditions but without the tested compounds. All experiments were repeated in triplicate. The IC_50_ values were determined from the inhibition curves using Curve Expert software (version 3.2) and the results were presented as the mean ± standard deviation (SD).

#### Kinetic study of enzyme inhibition

To evaluate the inhibition mechanism of AChE and BuChE, kinetic studies were carried out using compounds **6h** and **6f**, which showed the best in vitro AChE and BuChE inhibitory activity, respectively. The initial velocity (V) of the substrates hydrolysis was measured at different concentrations of acetylthiocholine iodide (ATCI) or butyrylthiocholine iodide (BTCI) (1.7–20 mM), in the absence (control) and presence of different concentrations of the compound **6h** (5, 10, 20, 40, and 80 μM) for AChE and different concentrations of compound **6f** (0.5, 1, 5, 10, and 20 μM) for BuChE. Then, the double reciprocal plot of 1/V versus 1/S was drawn to determine the inhibition type of AChE and BuChE. Data analysis was performed using Microsoft Office Excel 2016.

#### Antioxidant assay

The antioxidant activity of the synthesized compounds were determined based on the DPPH method. 2,2-Diphenyl-1-picrylhydrazyl (DPPH) and quercetin were obtained from Sigma Aldrich company. In this assay, quercetin was used as the control compound. Initially, stock solutions of the tested compounds with concentration of 40 mM were prepared in dimethyl sulfoxide (DMSO) solvent, and then the necessary dilutions were made in methanol to prepare the required concentrations ranging from 25 to 400 µM. This test was conducted in 96-well plates. Each well contained 180 μL of DPPH (110 μM) and 20 μL of different concentrations of the tested compounds, which were incubated for 30 min under dark condition. Then, the absorbance changes were measured at a wavelength of 517 nm using the Synergy HTX Multi-Mode Reader-BioTek. EC_50_ values for the compounds whose free radical trapping percentage was more than 50% at a concentration of 400 µM were obtained using CurveExpert software. Experiments were repeated three times for each concentration and the results were reported as the mean (m) ± standard deviation (SD).

### Molecular docking study

Molecular docking simulations were performed to study the ligand-receptor interactions using the Autodock Vina (1.1.2) package. The crystal structures of BuChE (PDB ID: 1P0I) was extracted from the RCSB Protein Data Bank and both water molecules and original ligand were removed from it. To validate the docking protocol, the re-docking process was performed and the RMSD value of 1.36°A was obtained, which confirmed the reliability of docking. The ligand structures were drawn, optimized and saved in mol_2_ format, which was then converted into the pdbqt format via Auto Dock Tools package (1.5.6)^38^. The grid box size was set at 56 × 56 × 56 ˚A and the center of grid box was placed at the co-crystalized ligand with coordinates x = 137.985, y = 122.725, z = 38.78 . The docking results were analyzed using discovery studio 2016 client.

### MD simulation

The Gromacs molecular dynamics program was utilized to investigated the molecular dynamics simulation of compound **6f** bound to human butyrylcholinesterase (PDB: 1P0I) on a Centos Linux server outfitted with GPUs. The atom types were defined, and the system dynamics were simulated using the Amber99sb force field. Topology parameters for compound **6f** were generated using the ACPYPE webserver. The solute (compound **6f**) within the human butyrylcholinesterase complex was placed in an octahedral box, and surrounded by TIP3P water molecules to solvate the protein complex. Na^+^ ions replaced water molecules to ensure system neutrality. NVT heating was then applied to the system. Following that compound **6f** was subjected to constraints in order to avoid significant conformational changes during the first stages of equilibration. The long-range electrostatic interactions were effectively handled using the particle-mesh Ewald (PME) method. An average pressure of 1 atm was balanced for the system. The NPT ensemble (constant number of particles, pressure, and temperature) was used to equilibrate the system for 500 ps. Typically, molecular dynamics simulations must run for a sufficient amount of time in order to provide trustworthy results. In order to find the optimal system equilibration point at a suitable temperature and pressure, the manufacturing MD run took 100 ns. Post-MD, the trajectory underwent correction for periodic boundary conditions. Atomic position variances from a reference frame were measured and the equilibrium time range inside the simulation was found using the root-mean-square deviation (RMSD). Finally, using the Gromos approach and a cut-off value of 0.2, the trajectory was clustered during the equilibrium time period. To evaluate molecular dynamics simulations and create visual representations of molecule structures and interactions, Discovery Studio Visualizer v20.1.0.19295 was used^39^.

## Conclusion

In this study, we focused on the synthesis of novel 2,4-disubstitued quinazoline derivatives using anthranilic acid, urea and various anilines. The chemical structures of the final compounds were established using spectroscopic techniques. The final compounds were evaluated as acetylcholinesterase (AChE) and butyrylcholinestrase (BuChE) inhibitors, as well as antioxidant agents. The synthesized compounds showed higher potency towards eqBuChE rather than eelAChE. Among all the synthesized compounds, **6f**, **6h**, and **6j** appeared as the most active and selective eqBuChE inhibitors. Compound **6f** (IC_50_ = 0.52 μM), with 4-Me substitution at the phenyl rings displayed maximum inhibitory activity against eqBuChE with a selectivity index (SI) of > 96 for eqBuChE over eelAChE. Interestingly, the eqBuChE inhibitory activity displayed by compound **6f** was 20 times more active than donepezil with IC_50_ value of 10.6 μM. The docking results demonstrate that the most active compounds **6f**, **6h**, and **6j** are well fixed in the active site of BuChE and interact with the critical residues. The MD simulation results indicated that compound **6f** interacts effectively with the hydrophobic pocket of the hBuChE receptor, which is in good agreement with the findings of docking studies. The obtained results suggest that compounds **6f**, **6h**, and **6j** are promising lead compouds for the further development of new potent and selective BuChE inhibitors.

### Supplementary Information

Supplementary Information.

## Data Availability

The data sets used and analyzed during the current study are available from the corresponding author upon reasonable request. We have presented all data in the form of Tables and Figures.
