# Supplementary material for: Synthesis, biological evaluation, molecular docking, and MD simulation of novel 2,4-disubstituted quinazoline derivatives as selective butyrylcholinesterase inhibitors and antioxidant agents
Source: Sci Rep. 2024 Jul 6;14:15577. doi: 10.1038/s41598-024-66424-z (PMC11227574; doi:10.1038/s41598-024-66424-z)
Supplement: Supplementary file 1 — Supplementary Information. [file 41598_2024_66424_MOESM1_ESM.docx]

**Synthesis, Biological Evaluation, Molecular Docking, and MD Simulation of Novel 2,4-Disubstituted Quinazoline Derivatives as Selective Butyrylcholinesterase Inhibitors and Antioxidant Agents**

Sara Sadeghian^a1^, Raziyeh Razmi^a1^, Soghra Khabnadideh ^a,b^, Mehdi Khoshneviszadeh^a^, Pegah Mardaneh ^a,c^, Arman Talashan^a^, Arman Pirouti^a^, Fatemeh Khebre ^a^, Zahra Zahmatkesh^a^, Zahra Rezaei^a,b^ ^[[1]](#footnote-1)^*

*^a^ Department of Medicinal Chemistry, School of Pharmacy, Shiraz University of Medical Sciences, Shiraz, Iran.*

*^b^ Pharmaceutical Sciences Research Center, Shiraz University of Medical Sciences, Shiraz, Iran.*

*^c^ Medicinal and Natural Products Chemistry Research Center, Shiraz University of Medical Sciences, Shiraz, Iran.*


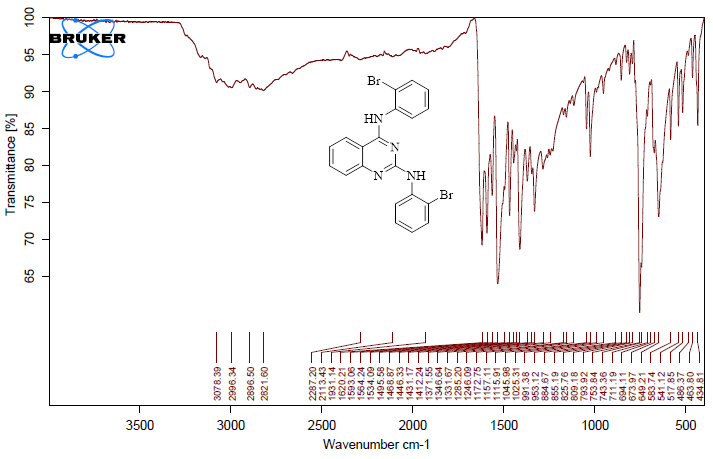


**Figure S1**. IR spectrum of **6a.**


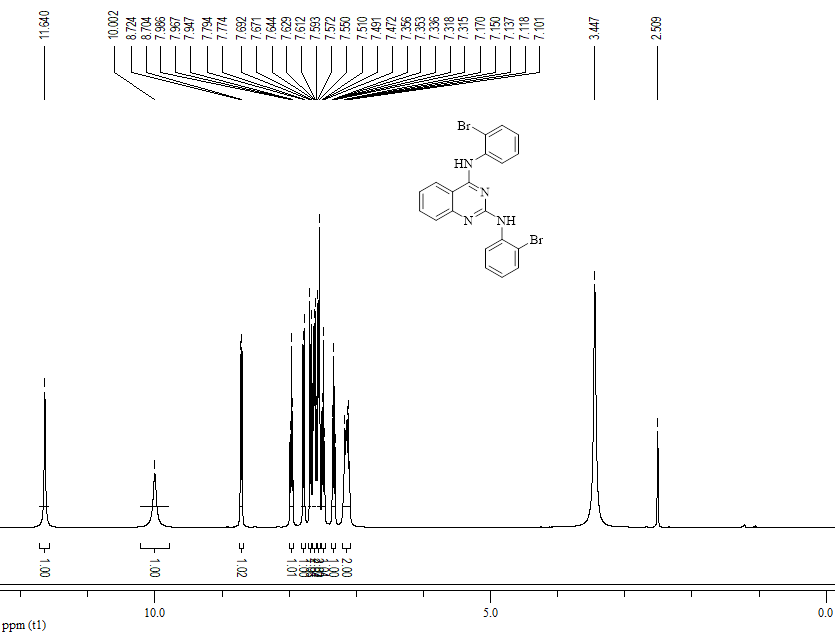


**Figure S2**. ^1^H-NMR spectrum of **6a.**

**
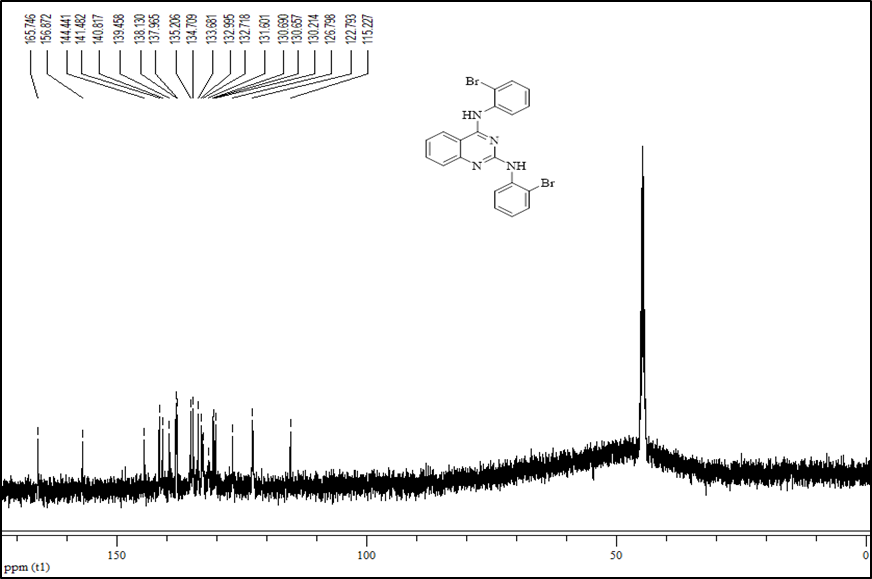
**

**Figure S3**. ^13^C-NMR spectrum of **6a.**


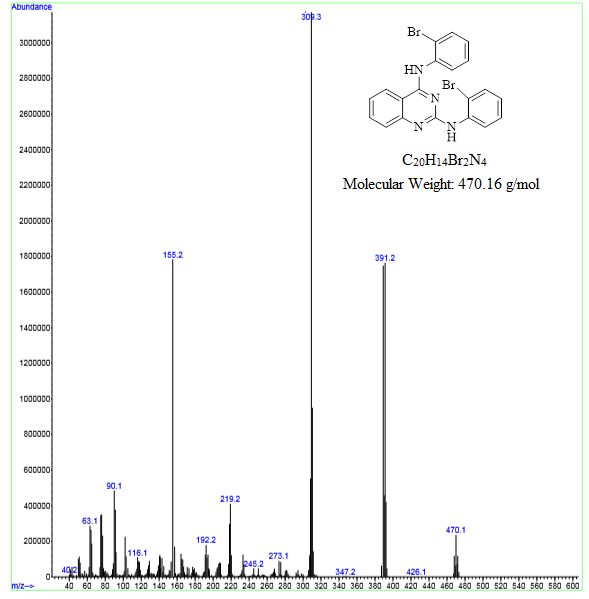


**Figure S4**. MASS spectrum of **6a.**


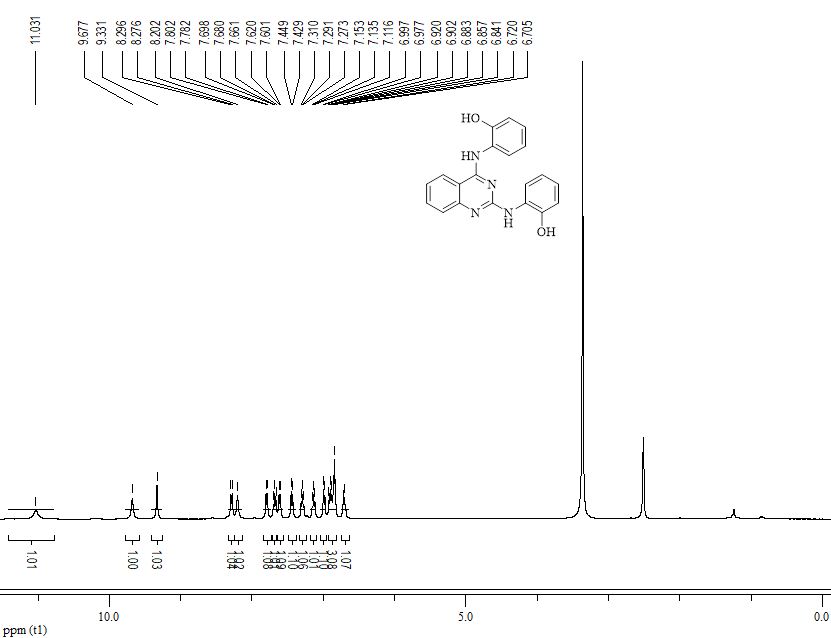


**Figure S5**. ^1^H-NMR spectrum of **6b.**


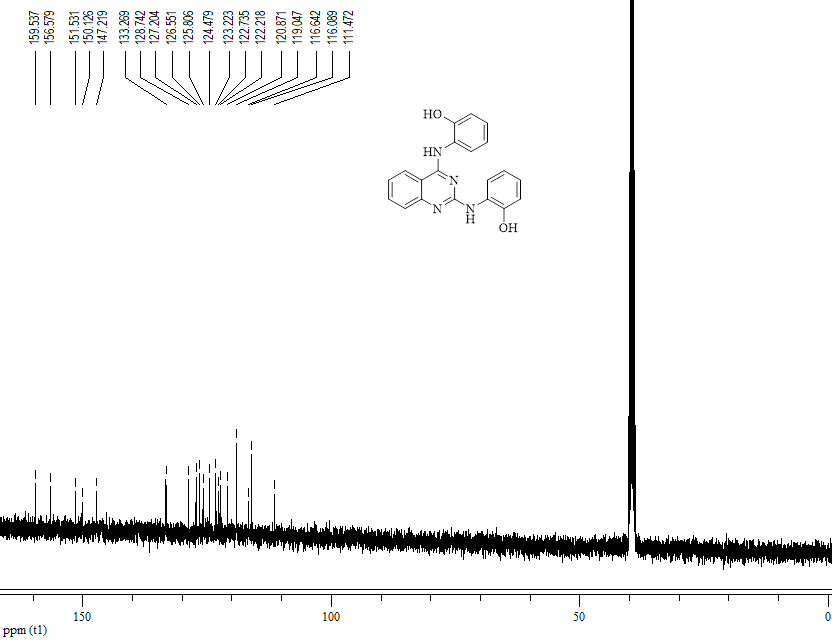


**Figure S6**. ^13^C-NMR spectrum of **6b.**


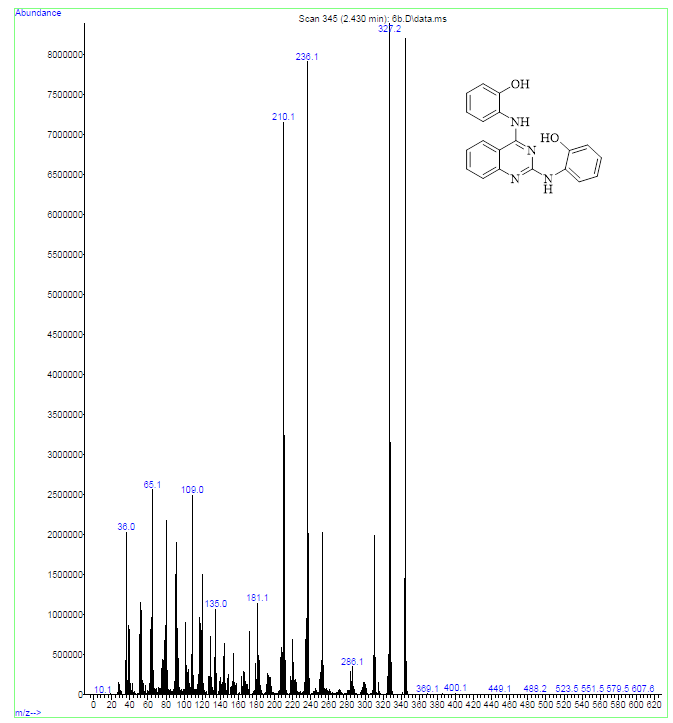


**Figure S7**. MASS spectrum of **6b.**


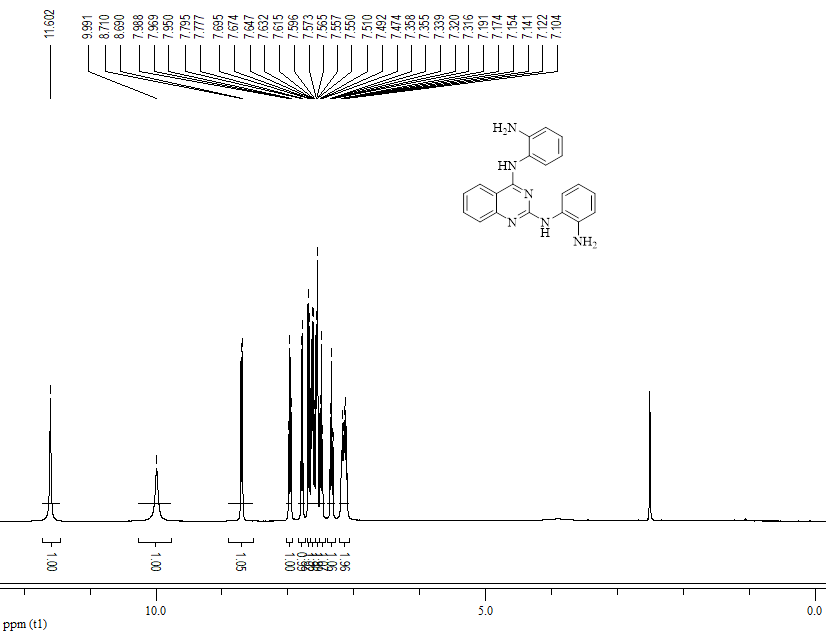


**Figure S8**. ^1^H-NMR spectrum of **6c.**

**
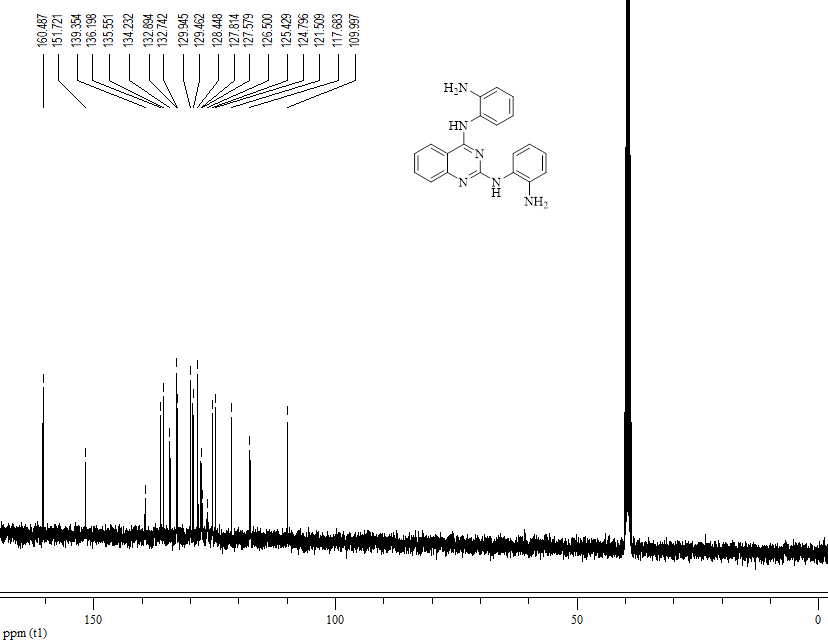
**

**Figure S9**. ^13^C-NMR spectrum of **6c.**


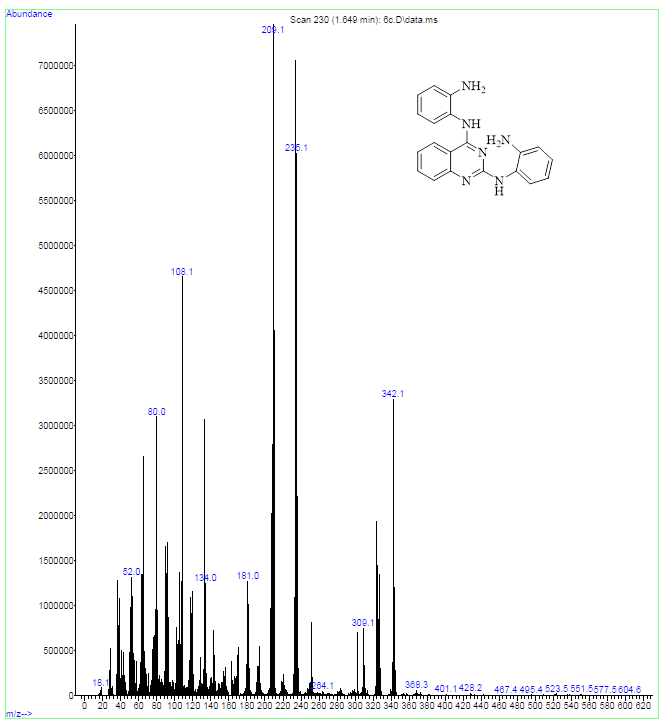


**Figure S10**. MASS spectrum of **6c.**

**
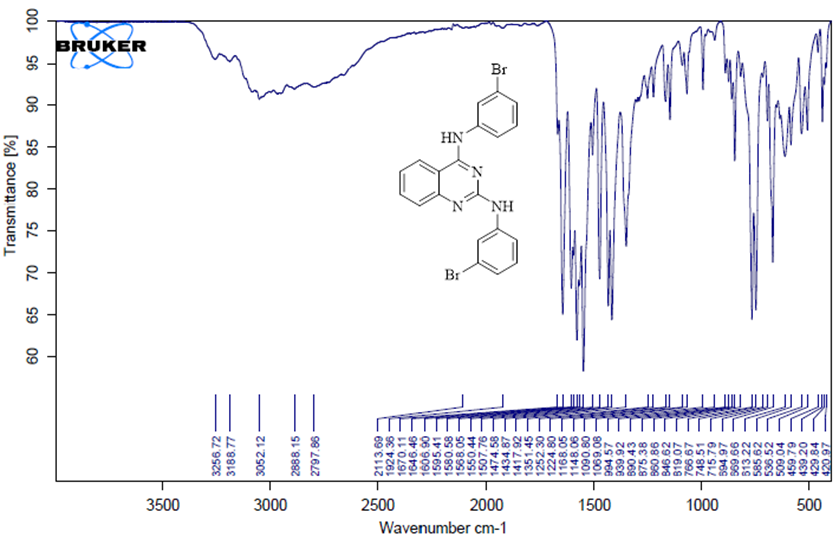
**

**Figure S11**. IR spectrum of **6d.**


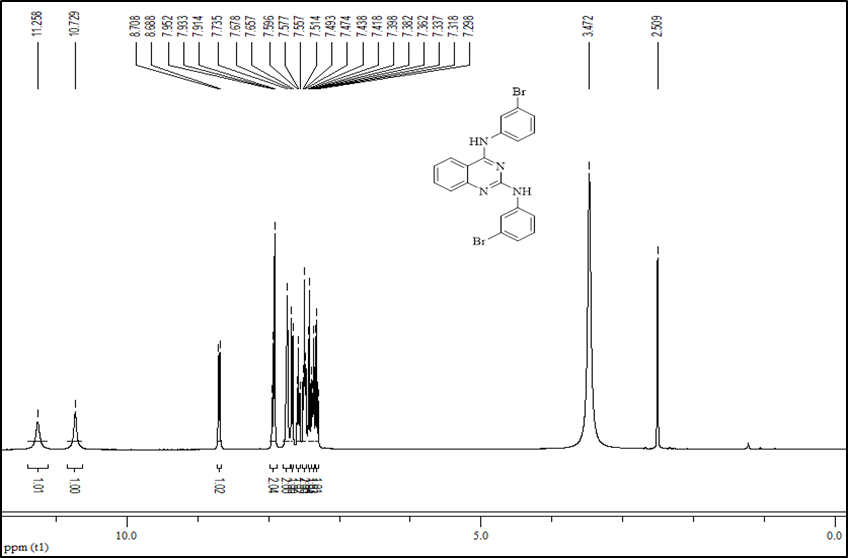


**Figure S12**. ^1^H-NMR spectrum of **6d.**


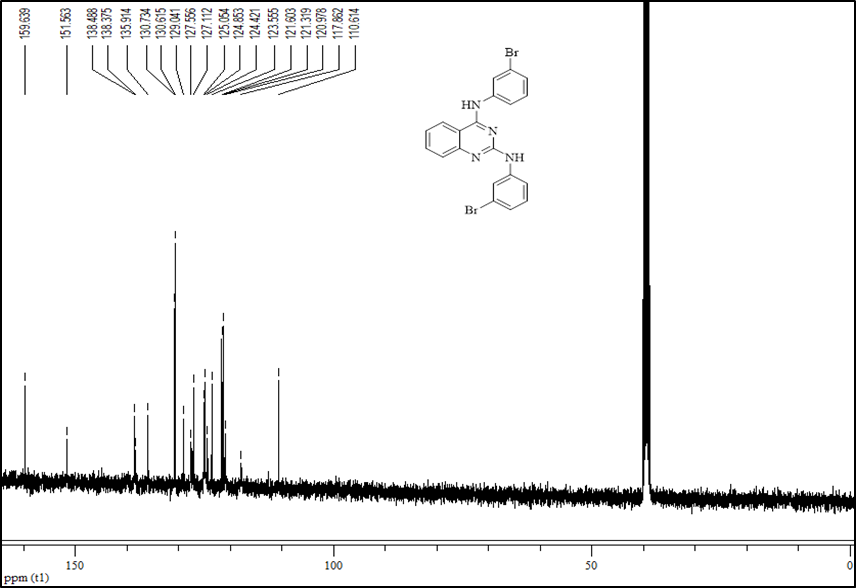


**Figure S13**. ^13^C-NMR spectrum of **6d.**


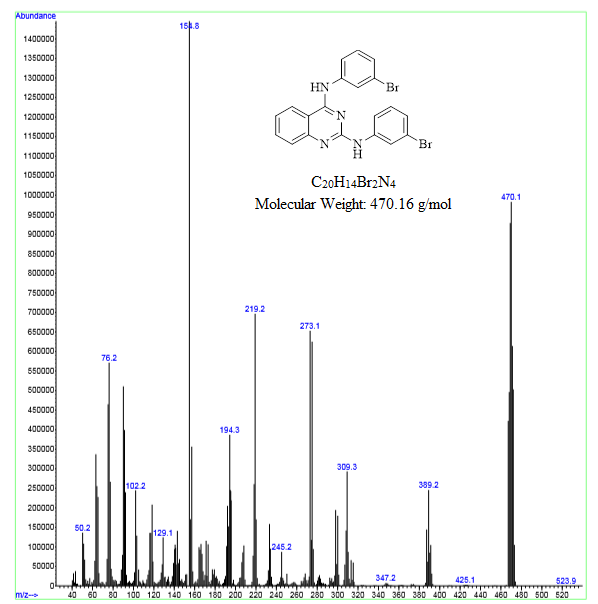


**Figure S14**. MASS spectrum of **6d.**

**
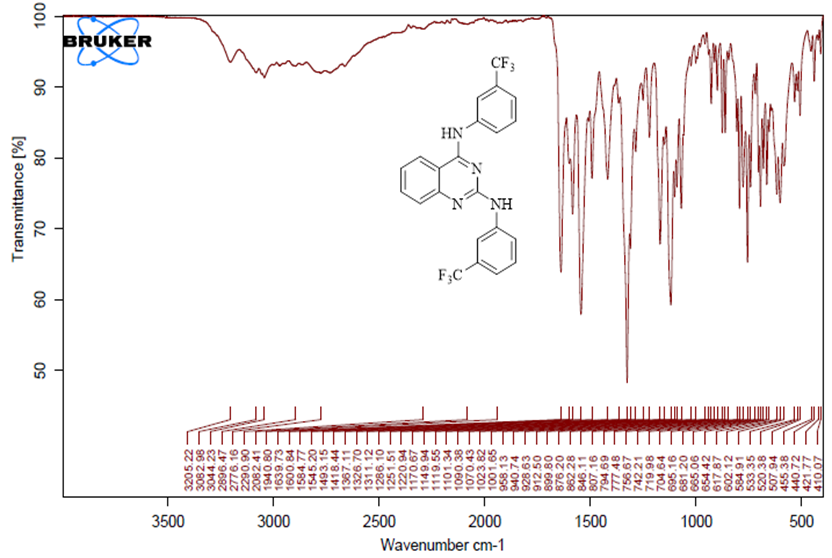
**

**Figure S15**. IR spectrum of **6e.**


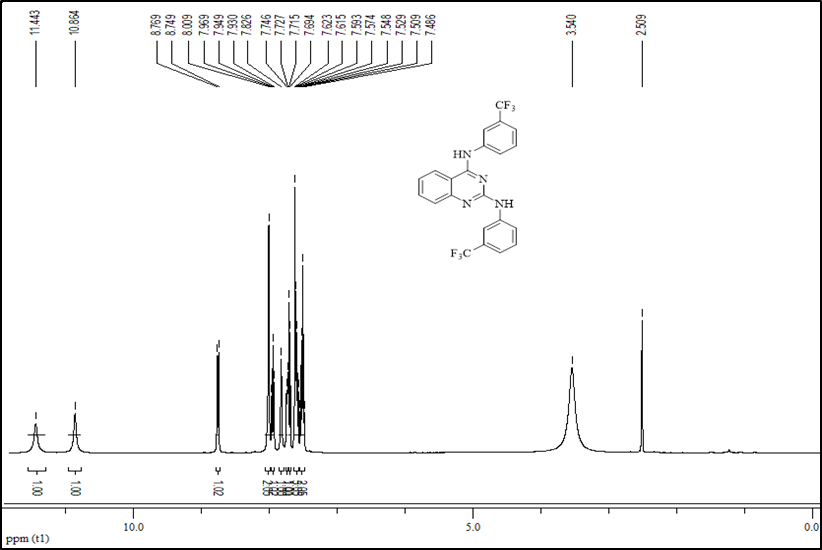


**Figure S16**. ^1^H-NMR spectrum of **6e.**


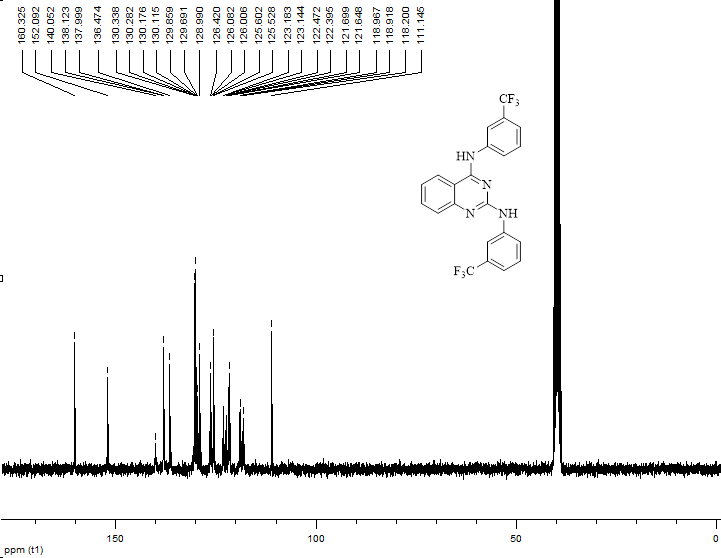

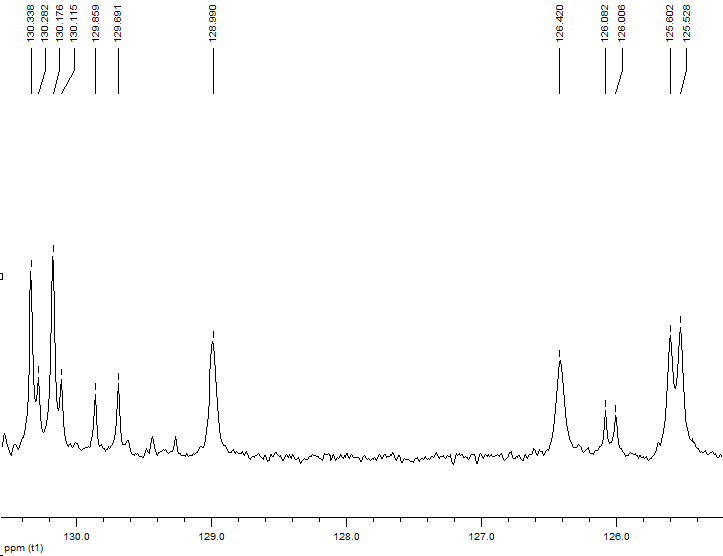


**Figure S17**. ^13^C-NMR spectrum of **6e.**


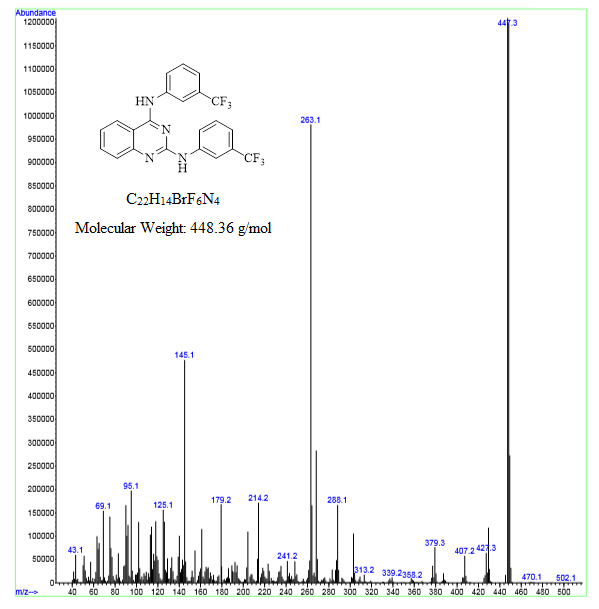


**Figure S18**. MASS spectrum of **6e.**


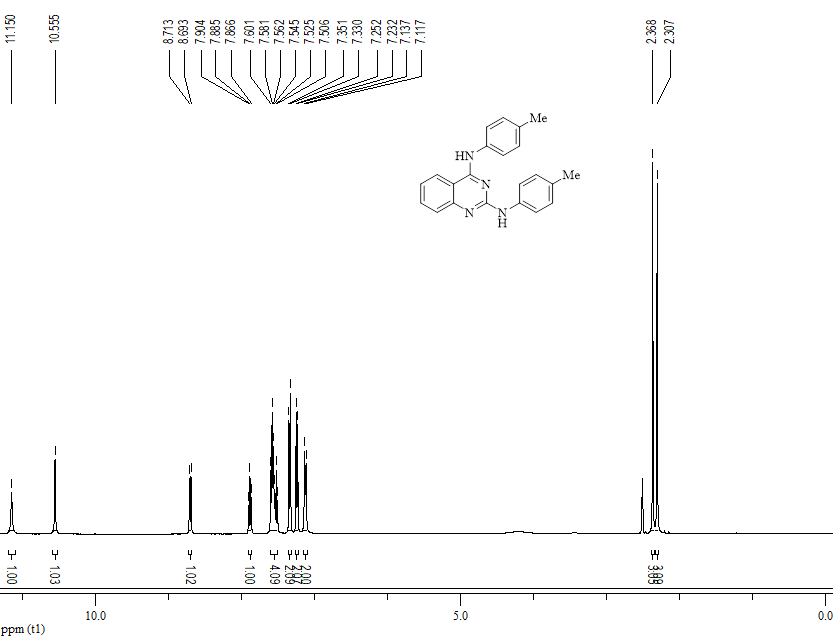


**Figure S19**. ^1^H-NMR spectrum of **6f.**


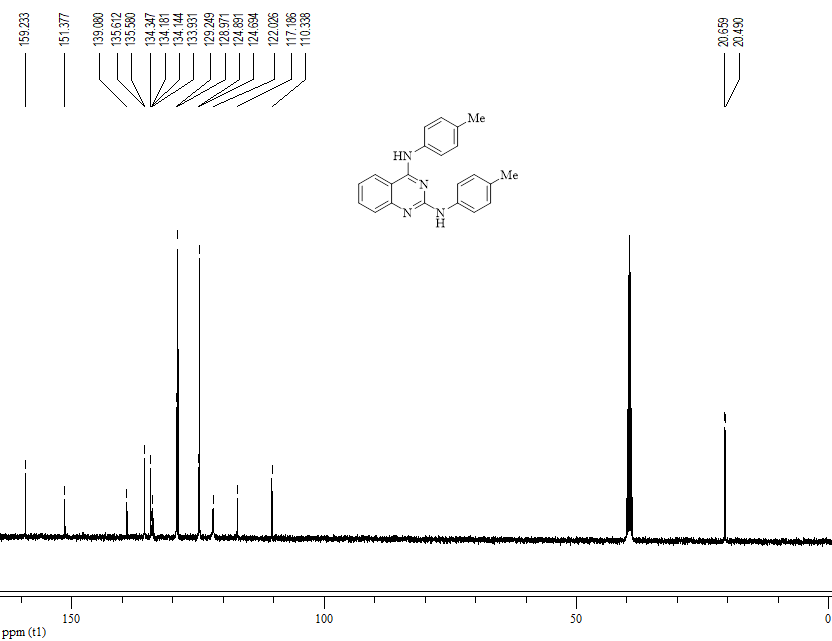


**Figure S20**. ^13^C-NMR spectrum of **6f.**


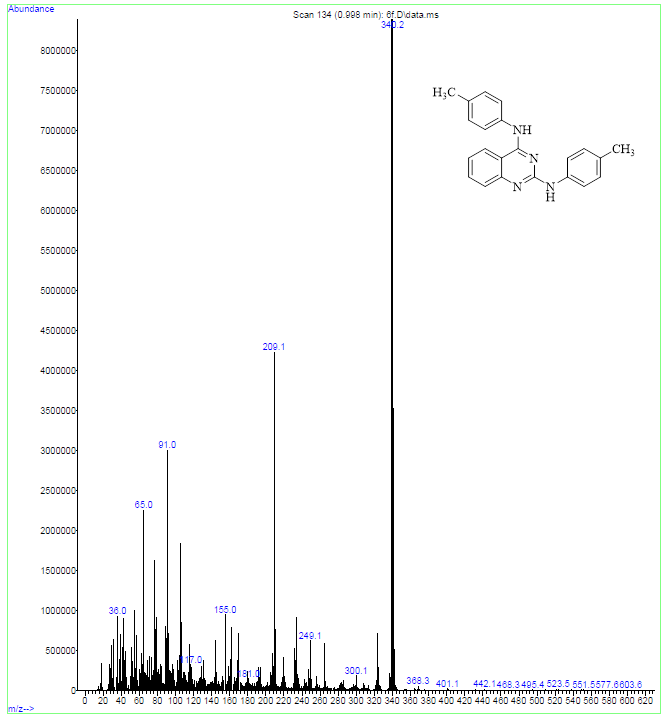


**Figure S21**. MASS spectrum of **6f.**


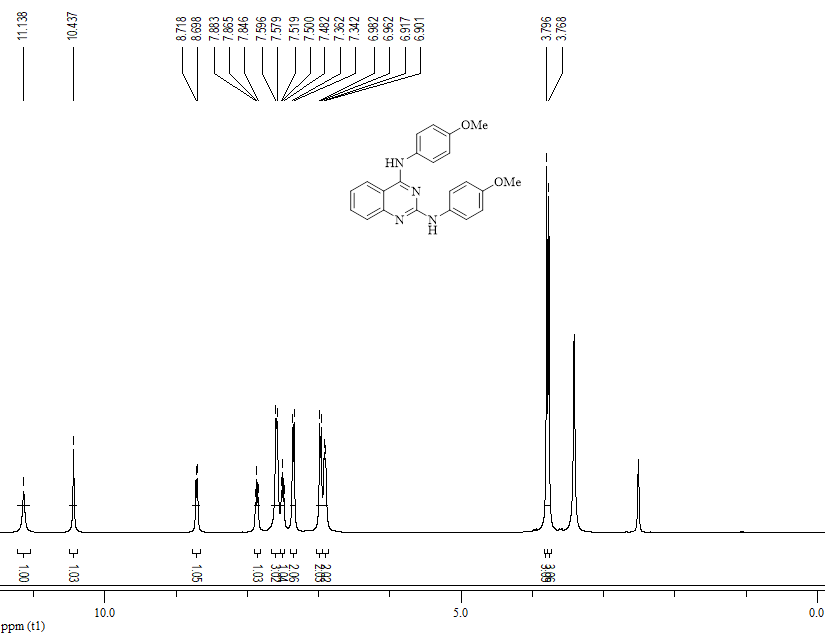


**Figure S22**. ^1^H-NMR spectrum of **6g.**


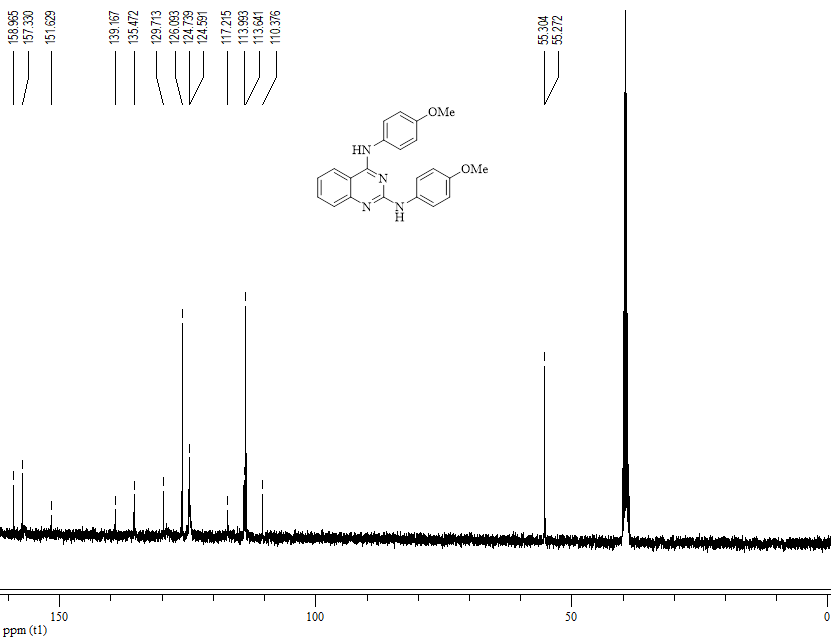


**Figure S23**. ^13^C-NMR spectrum of **6g.**


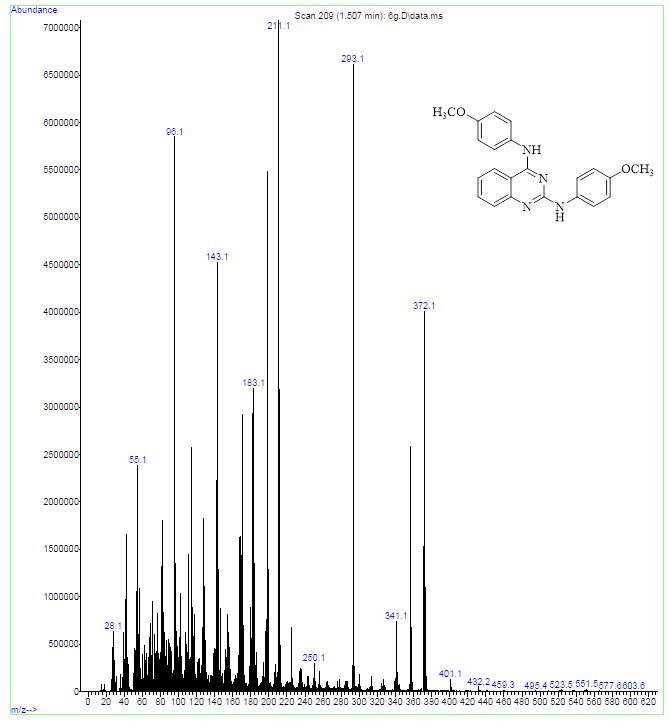


**Figure S24**. MASS spectrum of **6g.**


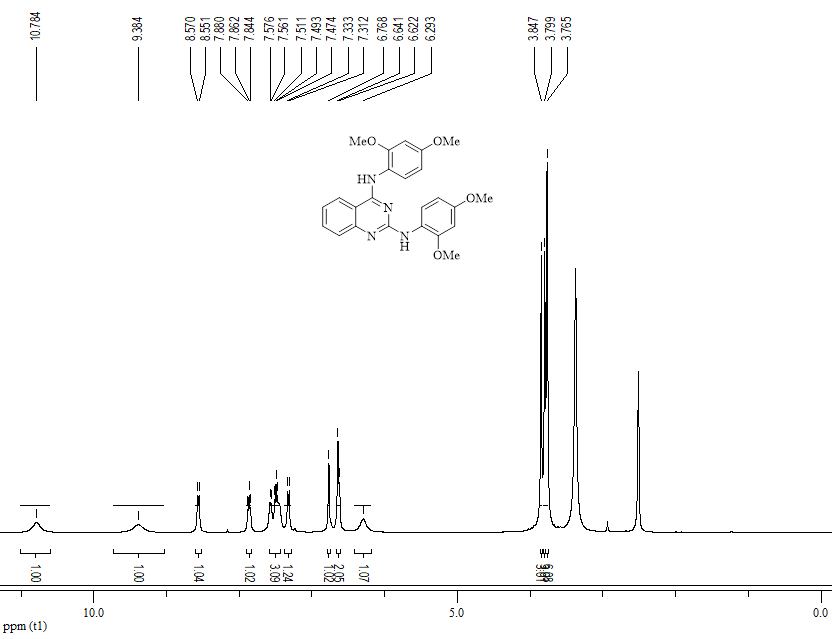


**Figure S25**. ^1^H-NMR spectrum of **6h.**


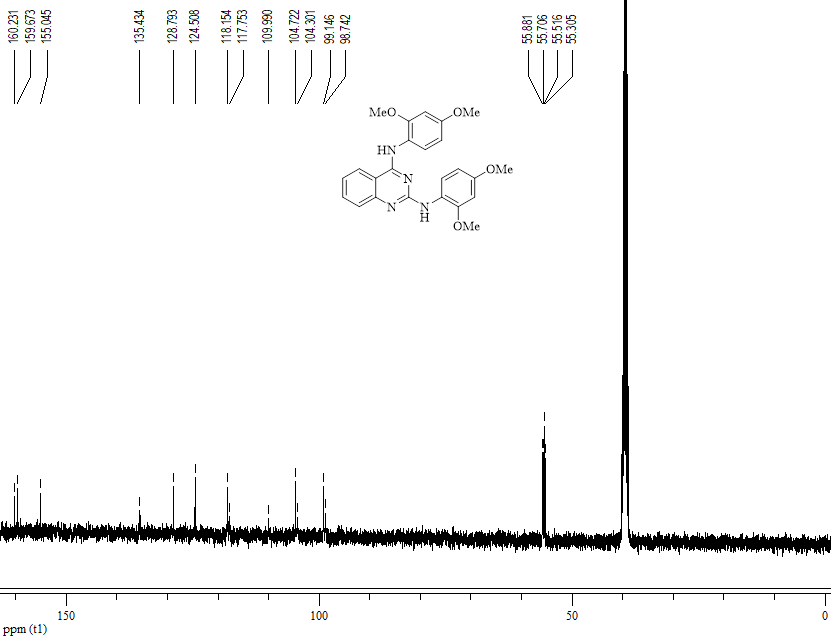


**Figure S26**. ^13^C-NMR spectrum of **6h.**

**
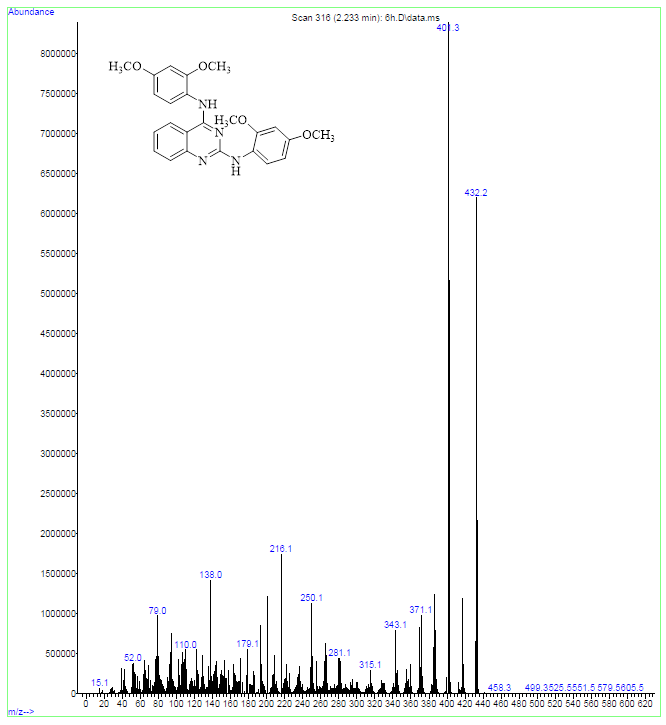
**

**Figure S27**. MASS spectrum of **6h.**


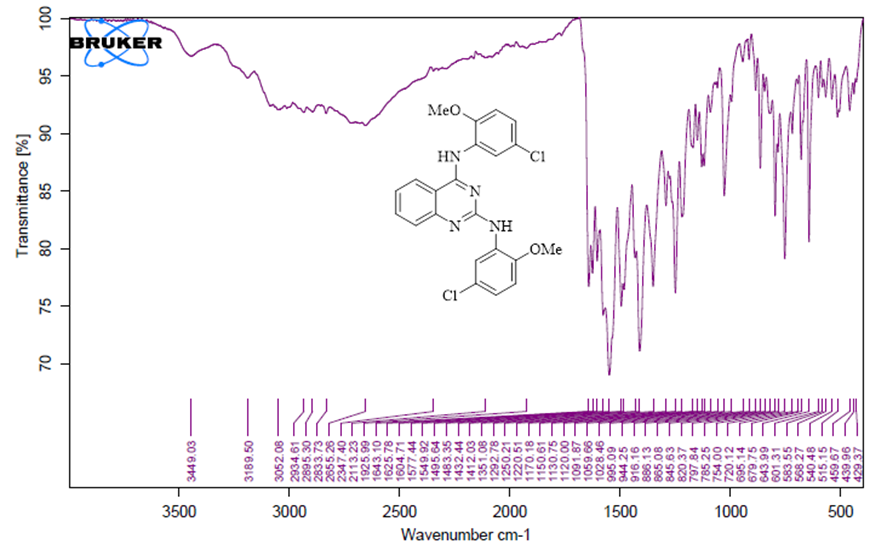


**Figure S28**. IR spectrum of **6i.**


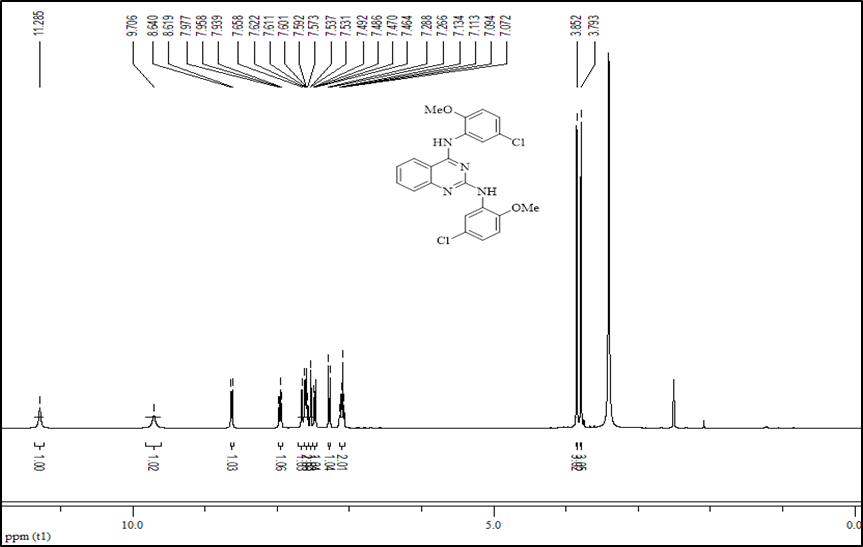


**Figure S29**. ^1^H-NMR spectrum of **6i.**

**
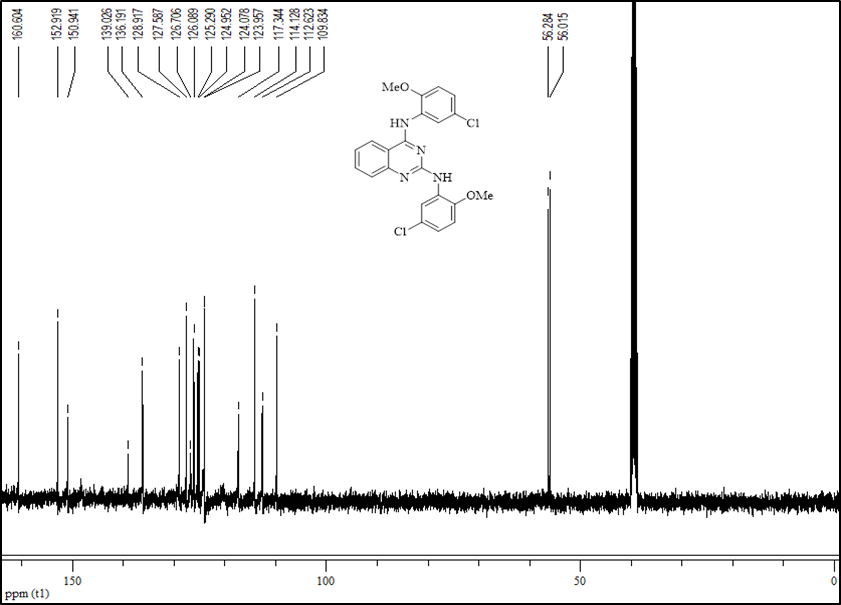
**

**Figure S30**. ^13^C-NMR spectrum of **6i.**


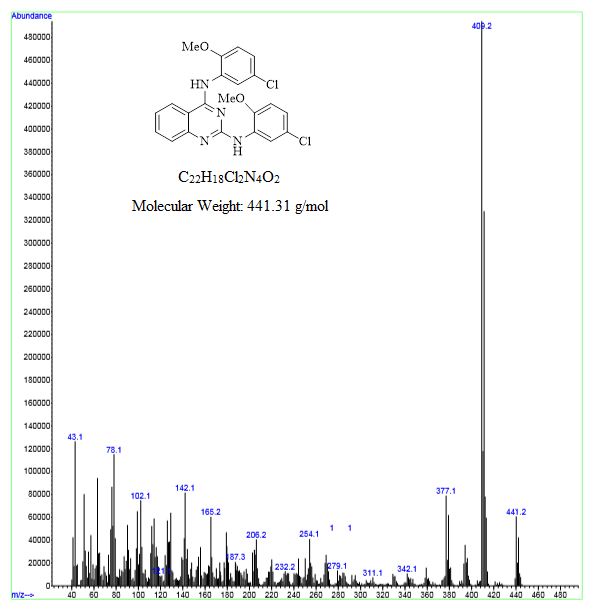


**Figure S31**. MASS spectrum of **6i.**


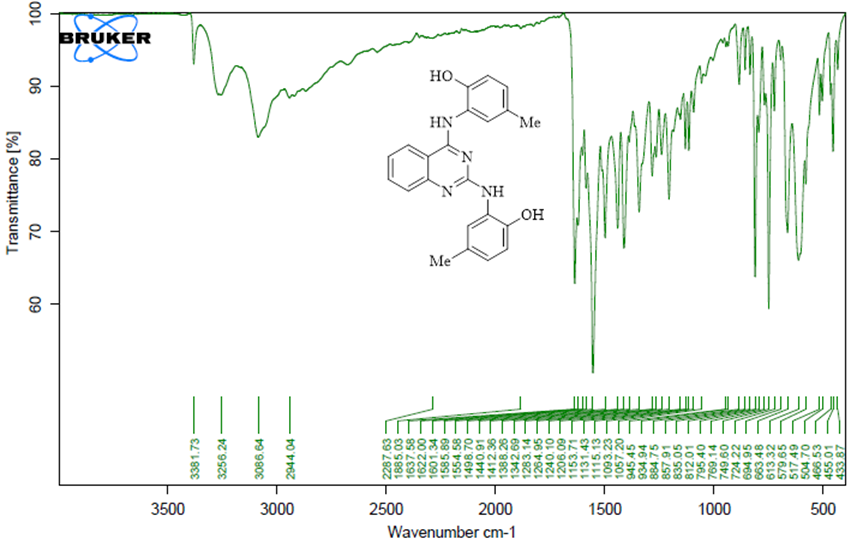


**Figure S32**. IR spectrum of **6j.**


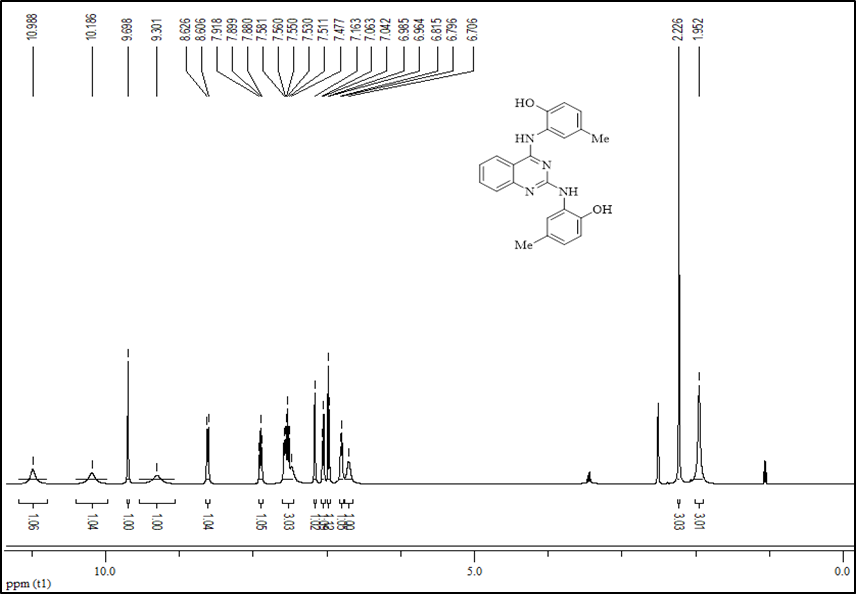


**Figure S33**. ^1^H-NMR spectrum of **6j.**

**
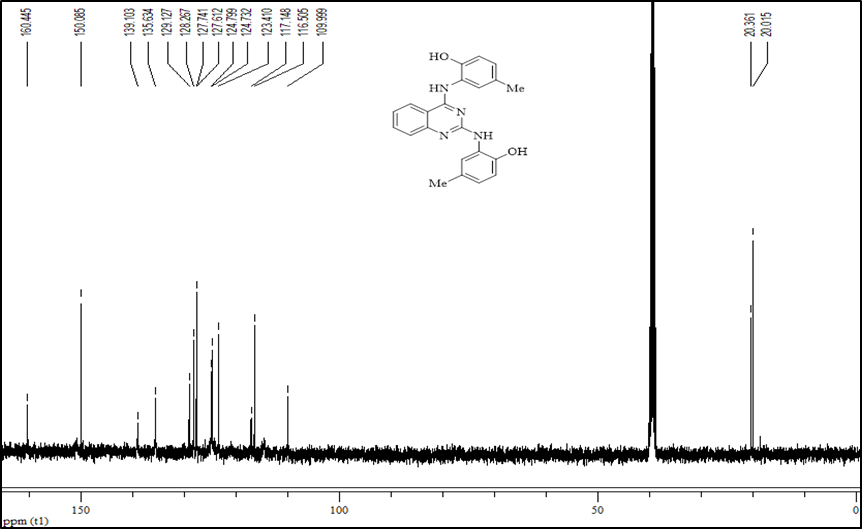
**

**Figure S34**. ^13^C-NMR spectrum of **6j.**


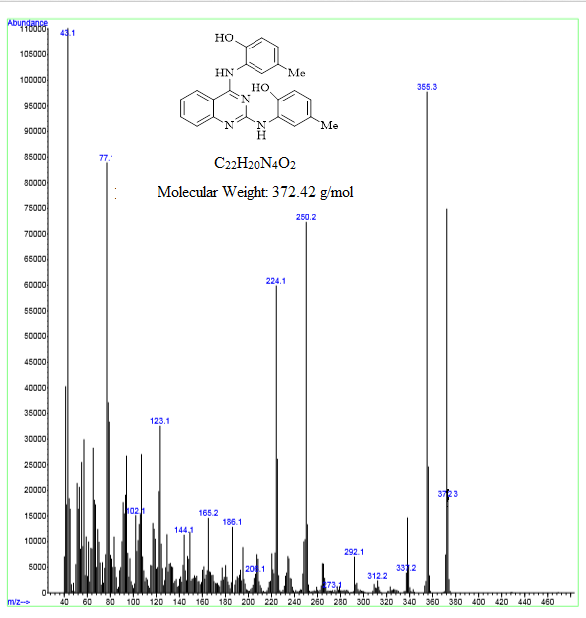


**Figure S35**. MASS spectrum of **6j.**

1. *Corresponding author: Zahra Rezaei, Pharmaceutical Sciences Research Center, Shiraz University of Medical Sciences, P.O. Box: 71345-1798 Shiraz, Iran. Email: [rezaeiza@sums.ac.ir](mailto:rezaeiza@sums.ac.ir)

   1: These authors contributed equally to this work. [↑](#footnote-ref-1)
